# Charting the Proteoform Landscape of Serum Proteins in Individual Donors by High-Resolution Native Mass Spectrometry

**DOI:** 10.1021/acs.analchem.2c02215

**Published:** 2022-09-08

**Authors:** Dario A. T. Cramer, Vojtech Franc, Tomislav Caval, Albert J. R. Heck

**Affiliations:** † Biomolecular Mass Spectrometry and Proteomics, Bijvoet Center for Biomolecular Research and Utrecht Institute for Pharmaceutical Science, University of Utrecht, Padualaan 8, Utrecht 3584 CH, The Netherlands; ‡ Netherlands Proteomics Centre, University of Utrecht, Padualaan 8, Utrecht 3584 CH, The Netherlands

## Abstract

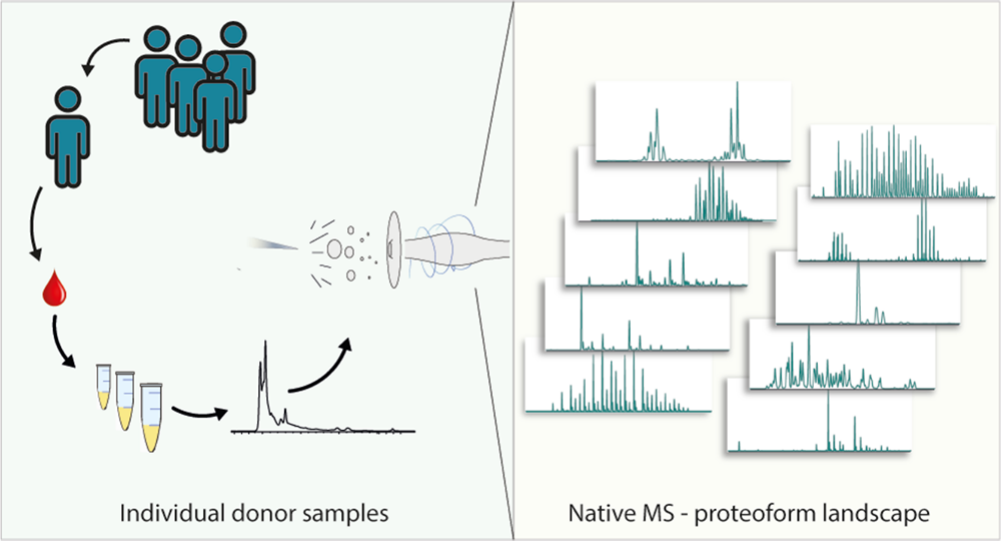

Most proteins in serum are glycosylated, with several annotated as biomarkers and thus diagnostically important and of interest for their role in disease. Most methods for analyzing serum glycoproteins employ either glycan release or glycopeptide centric mass spectrometry-based approaches, which provide excellent tools for analyzing known glycans but neglect previously undefined or unknown glycosylation and/or other co-occurring modifications. High-resolution native mass spectrometry is a relatively new technique for the analysis of intact glycoproteins, providing a “what you see is what you get” mass profile of a protein, allowing the qualitative and quantitative observation of all modifications present. So far, a disadvantage of this approach has been that it centers mostly on just one specific serum glycoprotein at the time. To address this issue, we introduce an ion-exchange chromatography-based fractionation method capable of isolating and analyzing, in parallel, over 20 serum (glyco)proteins, covering a mass range between 30 and 190 kDa, from 150 μL of serum. Although generating data in parallel for all these 20 proteins, we focus the discussion on the very complex proteoform profiles of four selected proteins, i.e., α-1-antitrypsin, ceruloplasmin, hemopexin, and complement protein C3. Our analyses provide an insight into the extensive proteoform landscape of serum proteins in individual donors, caused by the occurrence of various *N*- and *O*-glycans, protein cysteinylation, and co-occurring genetic variants. Moreover, native mass intact mass profiling also provided an edge over alternative approaches revealing the presence of apo- and holo-forms of ceruloplasmin and the endogenous proteolytic processing in plasma of among others complement protein C3. We also applied our approach to a small cohort of serum samples from healthy and diseased individuals. In these, we qualitatively and quantitatively monitored the changes in proteoform profiles of ceruloplasmin and revealed a substantial increase in fucosylation and glycan occupancy in patients with late-stage hepatocellular carcinoma and pancreatic cancer as compared to healthy donor samples.

## Introduction

Plasma and serum have become a rich source of biological information regarding both health and disease.^1,2^ The serum proteome shows rapid changes in protein abundance, glycosylation, and other post-translational remodeling in response to acute inflammation, cancer, and various environmental stimuli.^3^ As more than half of human serum proteins are glycoproteins, it is no surprise that several studies focus on changes in protein glycosylation. Furthermore, several FDA approved biomarkers are serum glycoproteins.^4^ Mass spectrometry (MS) is currently the analytical method of choice for protein glycosylation profiling. The analysis of released *N*-glycans represents the most common approach, enabling high throughput analysis of the glycome.^5^ Large-scale glycomic mapping of serum *N*-glycan profiles has enabled informative insight into glycosylation changes such as glycan branching, sialylation, and fucosylation, associated with many cancers.^6^ These analyses are regularly performed on whole sera, meaning that the proteins of origin of the detected glycans are often not known. With the advent of glycopeptide enrichment strategies,^7^ improved glycopeptide fragmentation schemes,^8,9^ and optimized bioinformatics, analysis of glycopeptides has become an important complementary mode of analysis.^10^ Most current serum proteome studies rely on these two methods to observe and monitor protein glycosylation. Although powerful, both methods can miss co-occurring post-translational modifications (PTMs), genotype variants, and other modifications not specified in the search parameters or removed during sample preparation. Glycan release-based methods primarily focus on *N*-glycans and largely ignore, for instance, *O*-glycans. In a glycopeptide-centric approach, glycosylation sites can be missed when no good peptide is available covering the site of interest. This highlights the need for complementary analytical approaches that can also be used to reveal unexpected PTMs.

For this reason, the analysis of intact proteins under native conditions is gaining traction as a valuable complementary approach. A single native spectrum provides a holistic overview of the proteoform mass distribution irrespective of the type of modification. The application of native MS for the analysis of protein glycosylation caught on with the introduction of high-resolution Orbitrap mass spectrometers with an extended mass range.^11,12^ High-resolution native MS has rapidly developed into a valuable tool for the characterization and quality control of glycosylated biopharmaceuticals.^13−17^ In addition, several applications to elucidate interesting biological phenomena governed by glycosylation have appeared. Notable examples include the observation that *N*-glycan branching and core fucosylation affect the binding of the anticoagulant warfarin to α-1-acid glycoprotein^18^ or the stabilization of the haptoglobin-hemoglobin complex formation by fucosylation.^19^ Additionally, recent work utilized native MS for elucidating changes in fetuin glycosylation in septic patients.^20^ Another study focused on the profiling of α-1-antichymotrypsin glycoproteoforms, demonstrating unique glycosylation remodeling in response to a septic episode that remained present weeks post sepsis recovery.^21^ The versatility of native MS is also demonstrated by work on different PTMs, like cysteinylation and oxidation in albumin.^22^

Most of these studies rely on purification of a single protein at a time, a process that can be tedious and costly. Access to multiple glycoproteins from a single donor represents an untapped treasure trove for characterization of glycosylation at the intact protein level. Here, we present a method allowing the rapid and efficient isolation of over 20 serum proteins, starting with approximately 150 μL of serum per donor. To gain more depth, we first depleted selectively, using a single column, three abundant serum proteins (i.e., IgG, serotransferrin, and albumin). Subsequently, selected IEX fractions were analyzed by high-resolution native MS. The presented method allows the in-parallel characterization of serum protein proteoform profiles from a single donor and is highly complementary to peptide-centric MS and glycan release approaches.

By using high-resolution native MS, we annotated a wide range of modifications on the studied glycoproteins, including but not limited to *N-* and *O-*glycosylation, cysteinylation, glycation, cleavage products of proteolytic activation, and single amino-acid variations induced by co-occurring genotypes. To demonstrate the capabilities of this approach, we focus on four glycoproteins varying widely in mass and proteoform features, namely, α-1-antitrypsin (A1AT), ceruloplasmin (CER), hemopexin (HPX), and complement component C3, but we also briefly discuss interesting features observed on other serum proteins. For the first time, we annotate the highly modified glycoprotein hemopexin, which contains up to five *N*-glycans and one *O*-glycan, albeit its serum proteoform profile appears to be rather simple. We could also monitor cleavages induced by protein activation using our approach, identifying simultaneously intact C3 and C3c, one of the stable cleavage products (in serum) after C3 activation. For A1AT, we monitored genetic polymorphism with apparent different serum abundances between the allele variants. Finally, we tested the applicability of the presented method to monitor one protein from multiple donors by quantifying the change in glycosylation in holo-ceruloplasmin under different physiological conditions.

## Experimental Procedures

Individual serum samples from six healthy donors were provided by Sanquin Research (Amsterdam, The Netherlands). Serum samples of diseased donors were purchased from Discovery Life Sciences (Columbus, OH, USA). Further details on the serum samples are provided in Table S1. To remove IgG, serotransferrin, and albumin, 150 μL serum aliquots were loaded on a 3-in-1 depletion column (HD-0301-10GFC, Good Biotech Corp., Taiwan) following the manufacturer’s instructions. This process was automated using an elution robot (Favonian, Apeldoorn, The Netherlands). These serum samples, depleted for three proteins, were fractionated over an ion-exchange setup composed of a tandem of cationic and anionic columns (PolyCAT A 204CT0510 and PolyWAX LP 204WX0510, PolyLC, USA) following a previously described method.^23^ Fractions were collected every 0.5 min (400 μL, 13–27 min). Annotation of A1AT, CER, HPX, and C3 was validated by comparing the retention times of commercial standards and their MS spectra. Protein fractions were buffer-exchanged into 150 mM AMAC (pH 7.5) by ultrafiltration with a 10 kDa cutoff filter. Sialidase (Merck, 10269611001 (Roche) from *Arthrobacter ureafaciens*) was used to remove sialic acid residues from several studied proteins, and PNGase F was used to cleave off *N*-glycans. Protein samples were analyzed on a modified Exactive Plus Orbitrap instrument with an extended mass range (Thermo Fisher Scientific, Bremen) as previously described^14^ using a *m*/*z* range of 500–15,000. Accurate masses of proteoforms were extracted by deconvolution of the raw native MS spectra to zero-charge spectra using PMi Intact Mass software (ProteinMetrics, version 4.1–4.3). Analysis of PTM composition after deconvolution was done manually. Glycan symbols and text nomenclature are based on the recommendations of the Consortium for Functional Glycomics.^24^ Quantification of CER proteoforms for comparison between donors was done using relative intensities. The difference between groups was statistically tested using Welch’s *t*-test. All statistics were performed in Graphpad Prism software (version 9.0.0). For bottom-up MS analysis, C3 protein samples were digested in solution for 30 min at 60 °C. Protein samples were digested for 4 h using trypsin at an enzyme-to-protein ratio of 1:100 and then overnight with a Glu-C enzyme at a ratio of 1:75, at 37 °C. All digests were desalted using a protocol as previously described^25^ prior to LC–MS analysis. Peptides of protein samples (100 ng) were separated and analyzed using an Ultimate HPLC nanoflow system coupled to an Exploris Orbitrap mass spectrometer (both Thermo Fisher Scientific, Bremen, Germany) as previously described.^25^ Raw data were searched manually to identify glycan fragment ions and confirm results obtained by data interpretation with Byonic software (v4.3.4, Protein Metrics Inc., San Fransisco, USA). Modifications included in the search were human *N*- and *O*-glycans, Glu- and Gly-pyrolysis, and Met- and Try-oxidation. A more detailed description of the used experimental procedures is provided in the Supporting Information.

## Results

### Fractionation and Analysis of Serum Glycoproteins

**Figure 1:**
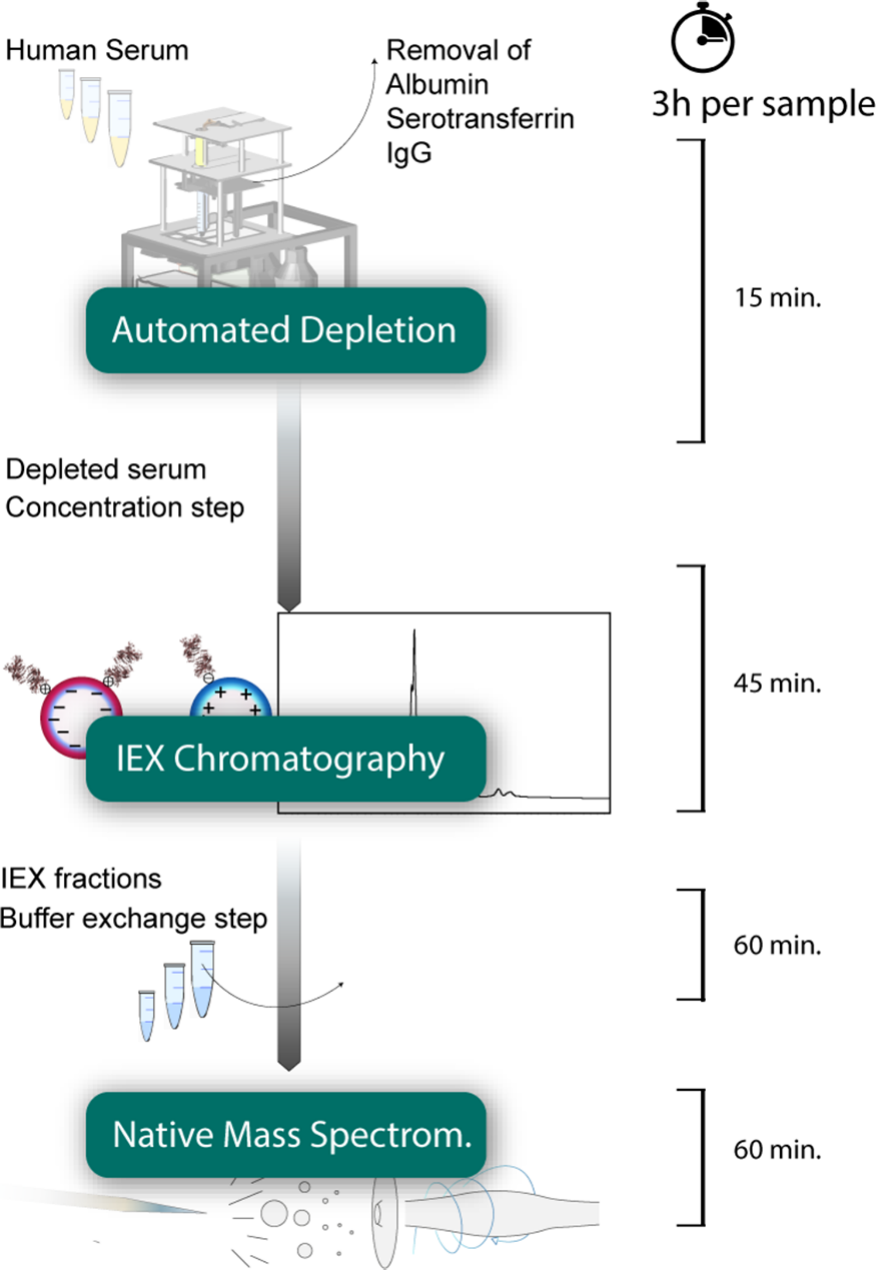
Fractionation and analysis of serum glycoproteins. Starting with 150 μL of serum, abundant serum proteins were depleted using an affinity column in an automated depletion step. The depleted serum sample was concentrated prior to fractionation using an ion-exchange column. Fractions of interest were collected and buffer-exchanged into a 150 mM AMAC buffer. Finally, the proteoform profiles of the intact proteins were analyzed by high-resolution native mass spectrometry, using direct infusion into an Orbitrap EMR. This approach takes about 3 h per analysis.

The primary aim of this work was to mass analyze in parallel several glycoproteins from minute amounts of serum acquired from individual donors and characterize their proteoform profiles using high-resolution native MS. We developed and automated a three-step purification method starting with the depletion of IgG, serotransferrin, and albumin using a commercially available depletion column. This step is followed by the separation and fractionation of the remaining serum proteins by IEX chromatography.^26^ Fractionated proteins are subsequently analyzed by direct-infusion under native conditions using an Orbitrap mass analyzer with an extended mass range (Figure 1).

**Figure 2:**
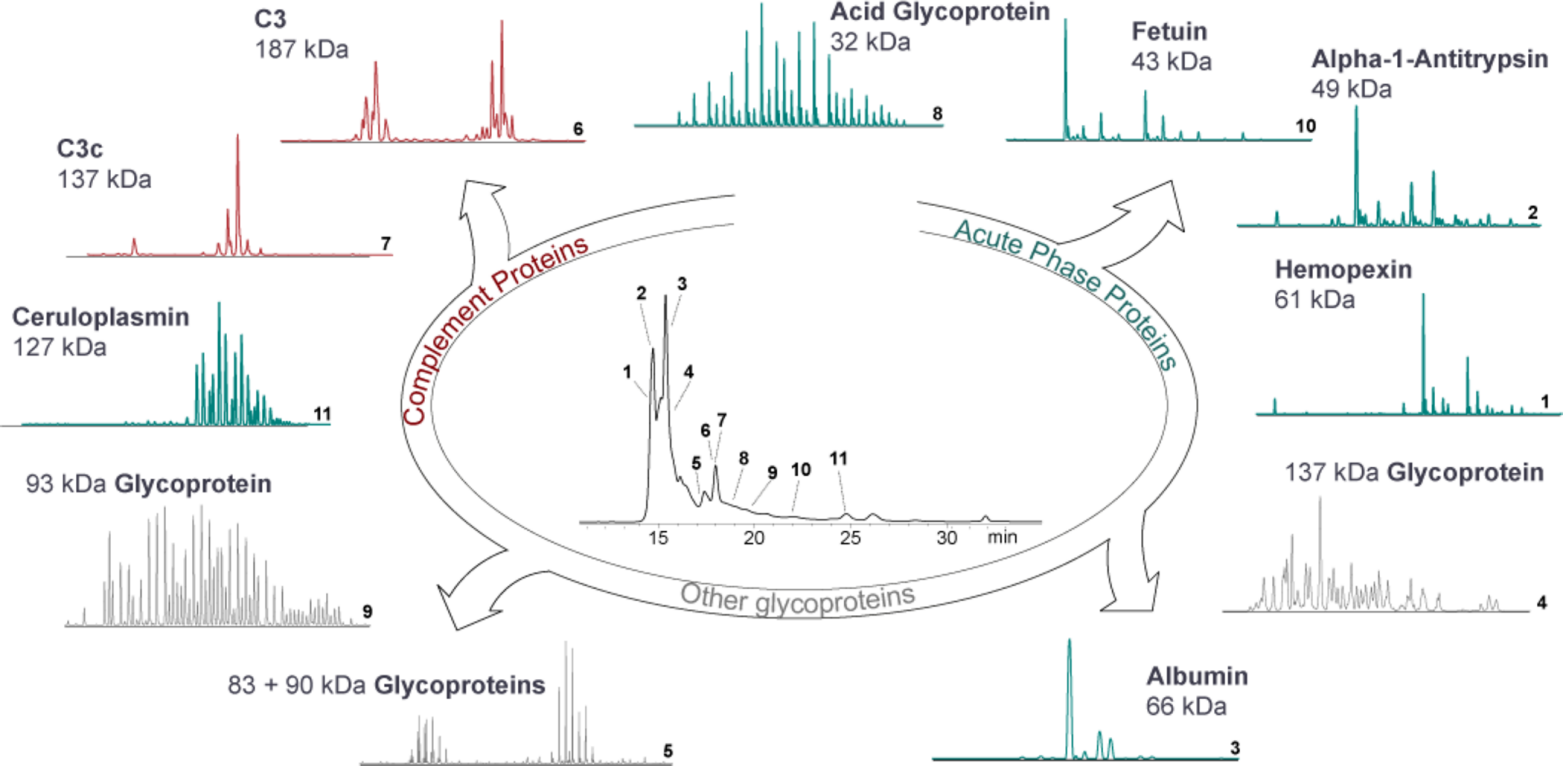
Charting the proteoform landscape of the serum proteome. A selection of isolated serum proteins analyzed by high-resolution native MS. Each individual fraction typically contains one or two proteins. Isolated proteins displayed extensive and variable proteoform profiles, caused largely by *N*- or *O*-glycosylation, other PTMs, polymorphism, or combinations thereof. The observed serum proteins spanned a wide mass range (30–190 kDa) and serum concentration range (0.2–50 g/L).

As we focus on abundant serum glycoproteins, we observed that 150 μL of serum was sufficient to obtain enough protein material for native MS analysis of each collected IEX fraction. The depletion step was added after preliminary IEX analysis revealed that albumin, IgG, and serotransferrin co-eluted with other proteins of interest. IEX chromatograms of serum show alike traces before and after depletion, albeit with the evident disappearance of albumin, IgG, and serotransferrin (Figure S3). We developed an in-house built robot to speed up the time-consuming step for this gravity-assisted column-based depletion. For the IEX chromatography, we used a gradient of 40 min, with most proteins eluting in a 14–27 min window (Figure S1). With the 40 min gradient and fractions taken every 0.5 min between 13 and 30 min, this automated setup allowed for the isolation and subsequent mass analysis of at least 20 unique serum (glyco)proteins in under 3 h. Each fraction was buffer-exchanged to aqueous ammonium acetate and subjected to high-resolution native mass spectrometry. Several illustrative zero-charge deconvoluted native mass spectra are depicted in Figure 2 to portray the wide variety of proteoform features of the analyzed serum proteins. Of note, we still detected some residual albumin in our analysis due to its high abundance in serum.

Although a native MS spectrum does not *a priori* enable the assignment of a protein identity, we used a three-step process to identify the proteins A1AT, C3, CER, and HPX. To achieve this, we compared the acquired native MS spectra with protein standards. We assigned mass shifts from the theoretical backbone mass to known glycosylation, either as reported by Uniprot or by following Clerc et al.^27^ Any remaining mass shifts could be assigned to disulfide bonds and other PTMs. An essential step in the data analysis was the deconvolution of the acquired spectra to zero-charge mass spectra. This deconvolution is not trivial, especially when multiple proteins and proteoforms provide congested spectra. Therefore, we used the PMi Intact Mass software to perform the deconvolution and checked the resulting data manually (Figures S4–S6). To analyze glycans and differentiate between glycan mass shifts of similar mass (i.e., fucoses and neuraminic acid), we used enzyme-based annotation strategies.^15^ In the case of other observed serum proteins, the MS spectra could be annotated by comparison with reported spectra, such as albumin,^22^ α-1-acid glycoprotein,^28^ and fetuin.^29^ As shown in Figure 2, we observed intriguing proteoform profiles of other glycoproteins that we did not identify or pursued further in-depth here. Instead, we focus on four selected proteins to showcase the complexity of proteoform profiles accessible with our method. The four selected proteins have been reported to portray aberrant glycosylation profiles in different diseases such as cancer,^30−32^ which we later use to demonstrate the potential of our approach.

### α-1-Antitrypsin: *N*-Linked Glycosylation, Cysteinylation, and Genotype-Dependent Abundances

**Figure 3:**
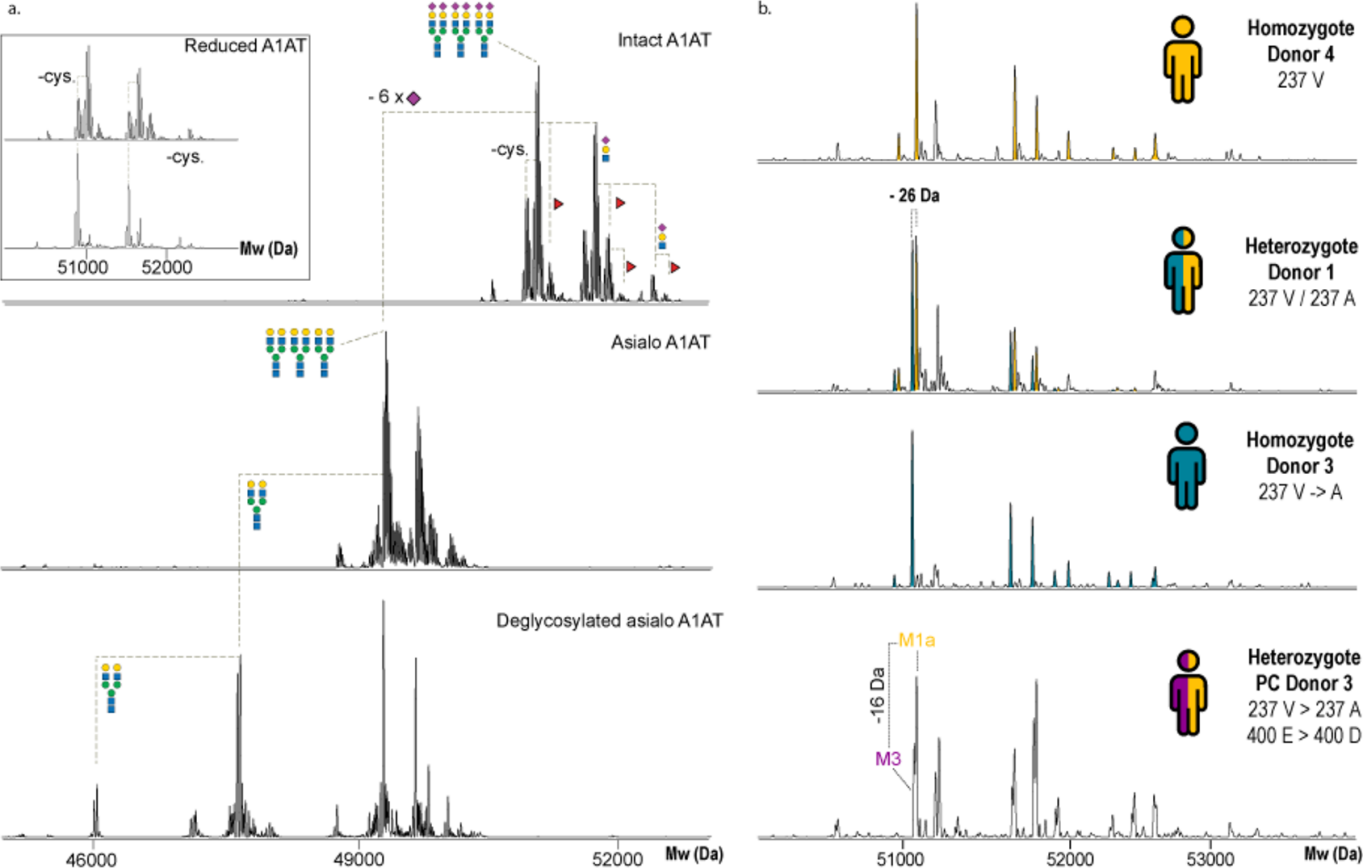
Characterization of the proteoform profile of α-1-antitrypsin. (a) From top to bottom, intact A1AT with annotated proteoforms revealing glycan antenna branching, fucosylation, and cysteinylation. An inset reveals the cysteinylation on A1AT, removed by TCEP treatment. Treatment with sialidase and PNGase F confirms the presence of three biantennary complex *N*-glycans on intact A1AT. This assignment also increases the confidence of the annotation of multiple monofucosylated glycoforms. (b) Deconvoluted native mass spectra of A1AT purified from three healthy individual donors (donors 4, 1, and 3 from top to bottom) and PC donor 3, a donor with pancreatic cancer. These A1AT proteoform profiles reveal four commonly occurring polymorphisms of A1AT. The proteoform profiles of these donors are qualitatively and quantitatively quite similar taking into consideration genotype induced mass shifts. The bottom spectrum obtained from PC donor 3 reveals proteoform profiles of alleles M1a and M3, observed as paired signals separated in mass by 16 Da. The serum abundance of these A1AT variants seems to be somewhat uneven.

The first serum protein whose proteoform profile we discuss is α-1-antitrypsin or A1AT, a 51 kDa acute phase glycoprotein. A1AT is a serine protease inhibitor known to bind neutrophil elastase. It plays an important role in the protection and function of the respiratory system. This is reflected by its usage in replacement therapy for people with A1AT deficiency. To get more insight into the proteoform profile, we first analyzed a commercially acquired A1AT standard. Figure 3a depicts the deconvoluted native MS spectra of this A1AT, before and after treatment with deglycosylation enzymes. For A1AT, we observed that treatment with sialidase led to a prominent down-shift in mass equal to the elimination of six sialic acid moieties (Figure 3a), in agreement with the expected presence of three complex biantennary *N*-glycans.^27,33^ Subsequent treatment with PNGase F removed up to two *N*-glycans (Figure 3a). This process allowed us to confidently annotate the glycosylation on A1AT (Figure S7). Our annotation is in good agreement with annotations based on earlier reported glycan- and glycopeptide data.^34,35^

The majority of serum A1AT was found to be cysteinylated, confirmed by TCEP treatment (Figure 3, top left inset) as a well-described feature for A1AT.^36^ Another distinct feature of A1AT is the partial truncation at the N-terminus (amino acids EDPQG), leading to additional proteoforms of the same glycan make-up, as also described by Kolarich et al.^37^ In all this data, we were able to dissect 13 A1AT glycoproteoforms (Table S3). Next, we analyzed A1AT extracted from individual donors and observed a variety of paired mass peaks originating from frequently occurring genotypes.^38^ A key example is the A1AT mutation of Val237Ala. For heterozygous donors, this leads to paired ion signals in the native mass spectra separated by the mass shift induced by the mutation (Figure 3b). Despite the proximity of these paired peaks, we could annotate the genotype of different donors. We identified four distinct genotypes in a sample set of 16 donors. We observed something peculiar for donors carrying both the V237A and E400D mutations on one allele (Figure 3b). Together, these two mutations induce a mass shift of 16 Da. These donors, being heterozygote, displayed a consistent unequal serum abundance of the M1a variant to the M3 variant. Whether this is true for larger sets of donors is something that needs to be further addressed but highlights how high-resolution native MS provides a manner to investigate small mass differences induced by polymorphisms.

### Complement Component C3: High Mannose Type *N*-Glycosylation and Proteolytic Processing

**Figure 4:**
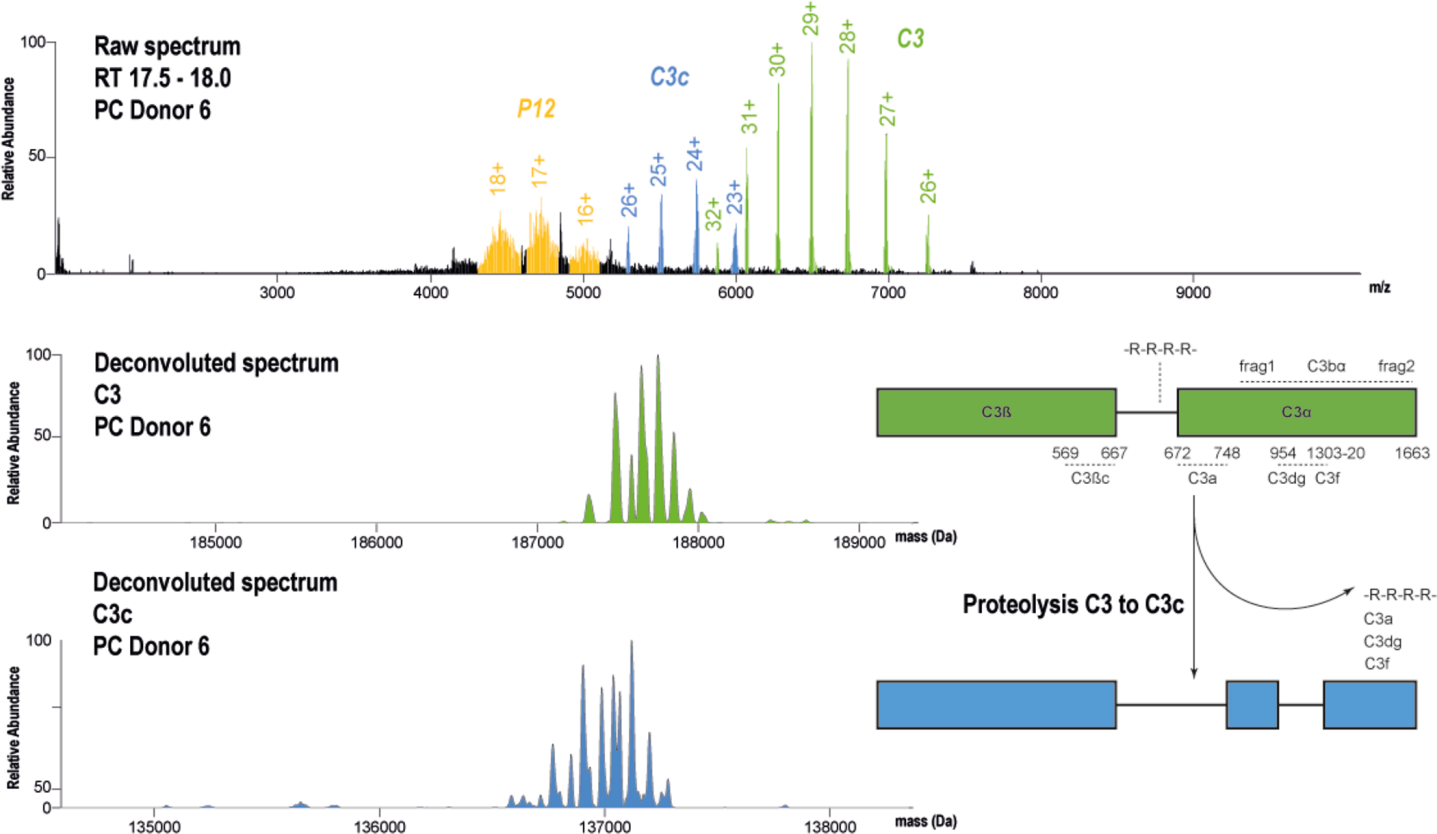
Proteoform profiles of serum C3 and C3c after fractionation by IEX. The top raw native spectrum reveals the co-elution of three proteins around 18 min retention time. Two proteins could be annotated as complement C3 (green, 187 kDa) and the fragment C3c (blue, 137 kDa). The inset schematic shows the processing and activation of complement C3 resulting in the formation of C3c. Important fragments and chains are annotated as well as the 4× Arg linker between the C3 beta and alpha chains.

The C3 protein is part of the complement system, playing a role in immunomodulation and a range of homeostatic processes^39^ and is present at a relatively high concentration in serum. The rather large 187 kDa C3 acute phase protein is highly reliant on its internal structural features, with a key role in the thioester bond between Cys 988 and Gln 991, essential for its activity. C3 harbors two high-mannose type *N*-glycans^40,41^ at Asn 63 and Asn 917. In our serum IEX chromatograms, C3 eluted around 18 min, displaying a rather simple proteoform profile (Figure 4). We recognized a co-fractionated protein of around 137 kDa protein displaying an alike proteoform profile and a highly heterogeneous unidentified glycoprotein protein (approximately 80 kDa) that we annotated P12. Treatment of this whole fraction with sialidase showed how the glycoprotein P12 shifted drastically in mass (Figure S8). In contrast, C3 and the 137 kDa protein did not exert noticeable mass shifts, confirming that there are sialic acids on P12, but no sialic acids on the (high-mannose) glycans of C3.

We could annotate C3 by comparing its native MS spectra with that of commercially acquired complement C3 (Figure S9). We attempted to match the observed mass of C3 to its backbone mass. For this, we took into account that C3 carries 13 disulfide bonds, the active site thioester, and the post-translational removal of four arginine residues on the intra-chain linker, providing a backbone mass of 184,302.6 Da. The remaining extra mass should then be attributable to attached glycan moieties (Table S4). We were able to annotate the mass of two oligomannose glycans with a combined number of in between 11 and 18 mannose moieties on the C3 standard. Although the proteoform profile of C3 looks relatively simple, we did observe peaks at +162 ± 5 Da mass gains, which we attributed to increases in additional mannose moieties. To confirm the presence of oligomannose glycans on complement C3, we deglycosylated C3 from donor 2 with PNGase F (Figure S10). We were unable to fully deglycosylate C3, but removing one oligomannose glycan left a less pronounced distribution of hexoses (+162 Da). This suggested that one oligomannose glycan remained, and we could match a glycan containing 6-mannoses with the theoretical backbone mass of C3. To corroborate our findings, we performed a glycopeptide analysis on fractions containing C3 from healthy donors, which revealed the occupation of glycosylation sites Asn 63 and Asn 917 (Figure S11) in donors 1 and 2, confirming that complement C3 carries two N-oligomannose glycans.

In the sera of donors’ complement C3 (Figure S9), the 137 kDa protein was annotated as C3c based on its similar proteoform profile compared to intact C3. An observed mass difference of 50,583 Da corresponds to the cleavage of C3a from the C3 alpha chain and consequent degradation of C3b into C3c.^42^ The presence of C3c in serum results from (possibly uncontrolled) complement activation. C3c has been proposed as a biomarker in heart failure^43^ and in several other diseases. In the collected fractions, C3, C3c, and protein P12 revealed variable abundance when comparing individual donors.

### Hemopexin: *O*- and *N*-Linked Glycosylation with Distinct Patterns

Next, we focused on hemopexin or HPX, whose main function in serum is to bind and transport free heme for heme and iron recycling. As such, HPX protects the body from oxidative damage that free heme can cause. In our IEX setup, HPX eluted quite early. Analysis by native MS revealed a relatively simple serum proteoform profile in a mass range between 58 and 63 kDa, while the protein backbone mass is just 50 kDa (Figure S12). Here, we provided the first full proteoform profiles of serum HPX and annotated the presence of four to five *N*-glycans and one *O*-glycan. These assignments were partly based on the fact that sialidase treatment removed 11 sialic acid moieties, hinting at maximally five complex *N*-glycans and possibly one sialylated *O*-glycan. These findings and annotations are in line with earlier glyco-peptide centric reports on HPX.^27^ Incubation with PNGase F further simplified the native mass spectra, leaving only the *O*-glycan attached. HPX also showed extensive fucosylation and glycan branching, which we could annotate after desialylation (Figure S13). Based on this data, we annotated 17 distinctive HPX glycoproteoforms (Table S5) encompassing the totality of all glycoforms on all glycosylation sites on the protein, with abundant glycoforms annotated in Figure S12.

### Ceruloplasmin: A Major Challenge in Proteoform Annotation

**Figure 5:**
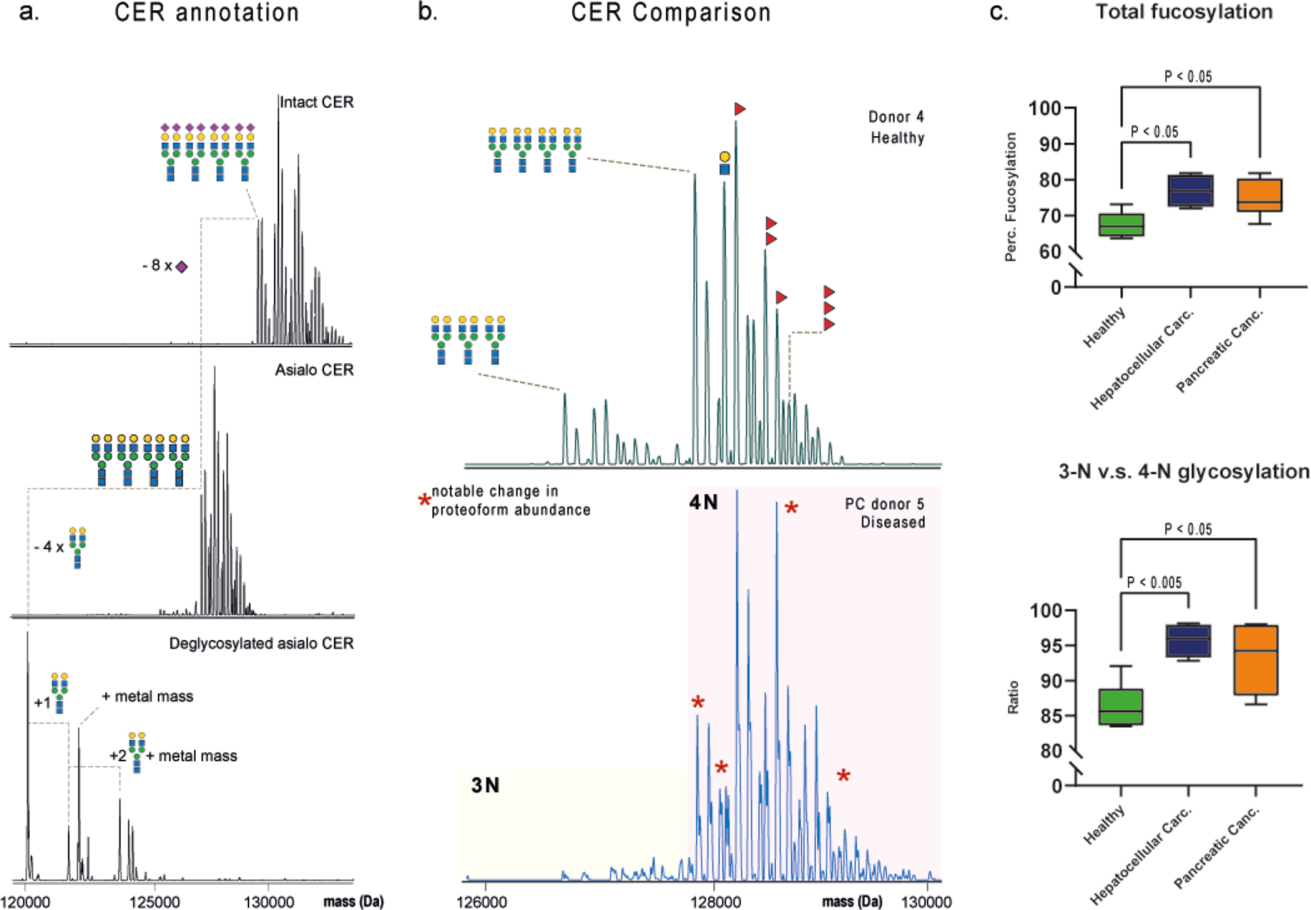
Ceruloplasmin proteoform profiles in health and disease. (a) From top to bottom, proteoform profile of intact serum-derived holo-CER, asialo CER, and deglycosylated CER, respectively. Deglycosylation shows that CER can be either fully or partially deglycosylated. A mass increase induced by the metal ions is observed when one or more glycan sites are occupied. Holo-Cer can be converted into apo-Cer by incubating the sample in AMBIC. (b) A side-by-side comparison of donors reveals disease-related changes in proteoform profiles. (c) Box plots depict the increase in fucosylation (top) in cancer and in the overall glycan site occupancy in cancer (bottom). Healthy donors (*n* = 6) were compared with donors with hepatocellular carcinoma (*n* = 4) and pancreatic cancer (*n* = 6). Statistical differences were calculated using Welch’s *t*-test.

Ceruloplasmin, or CER, is an ∼132 kDa acute phase glycoprotein that transports most of the copper in the human body (>95%). CER can carry six to seven copper ions per molecule.^44^ CER eluted relatively very late in our IEX fractionation and could be isolated to a high purity. We analyzed again by native MS serum CER, before and after incubation with deglycosylation enzymes. CER was subjected to sialidase and PNGase F (Figure S14). We identified the presence of four complex *N*-glycans, with a plethora of glycoproteoforms due to extensive fucosylation and glycan branching/elongation. We were able to annotate 32 distinct CER glycoproteoforms (Table S6). We were unable to directly compare the experimental mass of donor serum ceruloplasmin to the theoretical backbone mass even when including the glycosylations. Both in fractionated and commercially acquired standard ceruloplasmin, we observed an average mass excess of 409 ± 5 Da for all proteoforms compared to the corresponding theoretical masses. Based on the knowledge that ceruloplasmin carries coppers and appeared “blueish”, as a powder as well as in solution (2 μg/μL), we hypothesized the difference in mass to come from the expected six bound copper ions (6 × 63.45 Da) and one calcium ion (40.08 Da). The presence of six coppers and calcium on holo-CER has been well described,^44,45^ and together, they induce a mass gain in agreement with what is here observed. Intriguingly, upon full deglycosylation, this metal mass increment disappeared, and apo-CER could be observed as a single mass peak, corresponding to an expected backbone mass of 120,075 Da (taking into account 10 disulfide bonds) (Figure 5a). More surprising was the finding that in the partially deglycosylated CER with one glycan, we could observe the proteoform either with or without the additional metal ions. Upon occupation of two glycosylation sites, the metal ions were fully incorporated into the proteoforms containing two glycans (Figure 5a). These findings suggest that holo-CER is more resistant to PGNase induced deglycosylation or *vice versa*, and upon full deglycosylation of CER, nearly all metal ions lose (seemingly cooperatively) their binding affinity.

Next, we set out to confirm that the additional 409 Da in holo-CER came from the proposed metal ions. We suspected that AMBIC used as a solvent for deglycosylation could interrupt the interactions of CER with the metal ions. Therefore, we mass analyzed intact and deglycosylated ceruloplasmin following incubation in PBS and AMBIC. From the native spectra of these samples (Figure S15a,b), we concluded that incubation with AMBIC for 2 days is effective in removing all metal ions. When CER is incubated in PBS for the same time, this loss of metal ions does not occur. However, deglycosylation both in PBS and AMBIC for 48 h can lead to a complete loss of glycans and metal ions (Figure S16). Additionally, we observed that deglycosylation in PBS was less effective.

We further validated our findings that copper ions were present in serum CER by measuring the UV–vis absorption spectra of the same CER samples mentioned above (Figure S15c). Lyophilized CER and resuspended to a concentration of 2 μg/μL were of blue color, and we therefore expected absorption at 610 nm caused by the type I coppers in ceruloplasmin (blue copper).^44^ In agreement with the native MS data, CER incubated in PBS for 2 days still displayed an absorption peak around 610 nm, whereas this peak was absent when CER was incubated in AMBIC. In all analyzed donors, we observed mass corresponding to the holo-enzyme, harboring all metal ions, revealing that native MS can also be used to assess serum metalloproteins.

### Analysis of Individual Serum Protein Proteoform Profiles in Individual Donors

After in-depth characterization of holo-CER, we set out to probe proteoform variability in a small serum sample cohort (*n* = 16) to monitor changes in glycosylation between donors. We aimed to quantify the differences in glycan occupancy of CER as well as the level of fucosylation by comparing sera of people with late-stage hepatocellular cancer (*n* = 4), pancreatic cancer (*n* = 6), and healthy individuals (*n* = 6). These two outcome measures were chosen based on the prior hypothesis that increased fucosylation is associated with many types of cancer.^6,32^ We first set out to measure the CER proteoform profiles of all these donors (Supplement 1). We calculated the relative abundance of each glycoproteoform (Table S7) normalized to the most abundant peak in the proteoform profile. These relative abundances were used to quantify the level of fucosylation and the ratio of three to four *N*-glycans (Figure 5b,c). We observed an increase in overall fucosylation levels and glycosylation site occupancy for CER in the sera of cancer patients when compared to healthy donors. The ratio of occupancy of *N*-glycans was significantly higher in individuals with pancreatic cancer (*P* < 0.05) and hepatocellular carcinoma (*P* < 0.005) (Figure 5c). Fucosylation was also significantly increased both in the sera of donors with cancer (*P* < 0.05) when compared to healthy donors (Figure 5c). These data verify that a significant change in glycosylation patterns can be observed in response to (severe) diseases, although we note that our sample size is still rather small. Our data provide a proof of concept that native MS can also be used to determine such putative biomarker signals.

### Other Serum Proteins: A Range of Interesting Features in the Serum Glycoproteome

So far, we primarily focused our discussion on four serum proteins, although we fractionated and analyzed around two dozen of serum proteins (Table S2). We readily identified four more proteins based on similarity with previously reported native MS data: albumin, α-1-acid glycoprotein, fetuin, and plasminogen.

For completeness, all raw and deconvoluted spectra of proteins from all IEX fractions are displayed in Supplement 2 and sorted in order of their IEX retention times. Several interesting features were observed in their proteoform profiles. For instance, we detected plasminogen, the zymogen to plasmin that plays a role in fibrinolysis. Plasminogen (S2, protein #3) appeared as a partially, albeit highly phosphorylated protein and revealed two distinct proteoform profiles. These two distinct profiles were recognized as plasminogen type II, carrying both an *N*- and *O*-linked glycan and type I, lacking the *N*-glycan. As one of the few proteins highly phosphorylated in serum, its phosphorylation has been described.^46^ To our knowledge, we provide here the first overview of the full plasminogen proteoform profile. We also detected albumin, for which we could annotate glycation and cysteinylation (S2, protein #4), confirming earlier observations in serum.^22^ Also, α-1-acid glycoprotein (S2, protein #16) portrays a heterogeneous glycosylation pattern. We do not further discuss this protein as it has previously been extracted from serum using lectins, and its proteoform profile has been annotated.^28^ Other heavily decorated glycoproteins we observed had larger molecular weights. Notably, we fractionated and detected (so far) unannotated heterogeneous glycoproteins with Mws of around 88, 93, and 137 kDa (S2, proteins #13, #17, and #7, respectively). Though all complex in nature, annotation of charge states and even deconvolution of the spectra of these proteins were possible, albeit sometimes only after desialylation. In many of these data, we annotate mass shifts associated with glycosylation. Last, we also fractionated the smaller glycoprotein fetuin (S2, protein #18). Its glycosylation and phosphorylation have been extensively studied^20,29^ and compare well with the present data. We keep here the description and annotation of these other serum proteins concise but do want to iterate that our approach can chart quite a bit more of the proteoform landscape of the serum glycoproteome.

## Discussion

We developed a robust and sensitive analytical method for efficient isolation of multiple (glyco)proteins from low amounts of human serum circumventing the need for several affinity columns. Additionally, we used high-resolution native MS to characterize the proteoform profiles of these proteins in serum samples from single donors. Native MS provides a direct complete view of the proteoform profile. Although alternative approaches, such as glycopeptide analysis, are stronger in assigning glycans to specific sites and can be used to quantify site-specific glycans^47^ or describe glycoprotein meta-heterogeneity,^48^ native MS provides the most complementary protein-wide overview. We display the merits of our protein-centric approach by characterizing four acute phase glycoproteins isolated from serum in more detail. For most of these, we could annotate the full (and often complex) glycosylation patterns and monitor changes in glycosylation between donors and diseased states. Interestingly, we observed new features of these proteins, which were hitherto undescribed. These findings include the possibly unequal abundance of A1AT proteoforms originating from different alleles in heterozygote donors, the presence of metal ions in holo-CER and their role in resistance to PNGase F induced deglycosylation, the parallel monitoring of complement protein C3 and C3c, and the high occupancy of plasminogen phosphorylation, next to its *N*- and *O*-glycosylation. Last, our data provide for the first time the proteoform profiles of A1AT, HPX, C3, and CER, revealing that these profiles in serum are relatively simple, albeit different. Notably, C3 is decorated predominantly with two high-mannose *N*-glycans, whereas holo-CER is decorated predominantly with three to four fully sialyated complex *N*-glycans.

An additional advantage of native MS is that only a few sample preparation steps are required, which all can introduce biases. For instance, we observed the cysteinylation state of A1AT, which would be easily missed with standard peptide-centric proteomics approaches where typically reduction and cysteine alkylation are included. Furthermore, our protein-centric approach allowed us to identify A1AT polymorphisms that could also be easily overlooked with peptide-centric approaches when only one genotype of A1AT would be included in the search database (i.e., Swissprot). In HPX, native MS facilitated a straightforward annotation of its glycosylation profile including the *O*-glycan, using sialidase and PNGase F. The use of these latter two enzymes helped us in the annotation of all serum glycoproteins. In our hands, the sialidase treatment works very well, leading to the efficient release of all sialic acid moieties. However, treating glycoproteins with PNGase F under native conditions often only leads to the partial deglycosylation of the treated serum proteins. This is possibly undesired, although it may also shed light on which sites are protected. This became apparent in treating holo- and apo-CER with PNGase F, where we observed that holo-CER was more resistant to *N*-deglycosylation, whereas apo-CER could be deglycosylated easier.

Evidently, a downside of our approach, as is true for many proteomics approaches, is that we are biased toward the most abundant proteins in serum. However, we were quite pleased to be less biased in detecting high molecular weight proteins, as illustrated by in particular C3, which has a high mass of around 190 kDa. We feel that the consistent co-elution of C3 with its stable activation cleavage fragment C3c may be an excellent target for further in-depth characterization and potentially used to study complement activation. Another downside of our approach is that although we recorded the high-resolution native MS spectra of at least 20 different serum proteins, we annotated at present only eight of these (Table S2). We likely could further study and annotate these proteins with more efforts, using approaches such as peptide-centric proteomics, top-down proteomics, and glycomics.

Still, given the amount of different new features we observed by selecting only four proteins, a treasure trove of in-depth protein information is ripe for the picking, facilitated by the effective separation of multiple serum proteins. An interesting new development with native MS in this regard is the online coupling of MS with native chromatography methods, such as size exclusion chromatography^49,50^ or ion-exchange chromatography. We suggest for future development improving the efficiency of the current method by adding an online setup to improve sample throughput. Additionally, the total protein count could likely be optimized. In fact, we have observed proteins that only fractionated in single donors. Certain proteins could be increased in abundance under certain physiological conditions and enriched by applying a secondary separation step after ion-exchange chromatography.

In agreement with literature,^51,52^ we observed consistent increases in fucosylation and glycan occupancy in patients suffering from pancreatic or hepatocellular cancer. Earlier glycomics studies focused more on total fucosylation or changes in glycosylation but were performed on larger datasets, implying that these changes could be used as a biomarker. Here, we observe in a small cohort alike changes in abundances when comparing single individuals.

In summary, the approach presented here opens avenues for glycoproteoform profiling and associating changes in glycosylation, other co- and post-translational modifications, and polymorphism to various pathophysiological states. Fundamentally, the reported method can serve as a tool for detailed characterizations of serum glycoproteins to better understand their functions as well as their PTMs and glycosylation, their role in diseases, and for the discovery and quantitative measurement of serum protein biomarkers.

## Notes

All mass spectrometry data are available via the PRIDE repository (ProteomeXchange Consortium) with the identifier PXD033978.

## Supporting Information Available

Supporting tables with serum donor information, overview of observed proteins, and proteoform annotations, including peak abundances for CER comparison (XLSX)
Additional results including IEX chromatograms and native and bottom-up MS spectra used for annotation (PDF)
Supplement 1: native MS spectra of ceruloplasmin measured in all donors (PDF)
Supplement 2: native MS spectra of all fractionated proteins in order of retention time (PDF)
