# Supplementary material for: Charting the Proteoform Landscape of Serum Proteins in Individual Donors by High-Resolution Native Mass Spectrometry
Source: Anal Chem. 2022 Sep 8;94(37):12732–41. doi: 10.1021/acs.analchem.2c02215 (PMC9494300; doi:10.1021/acs.analchem.2c02215)
Supplement: Supplementary file 2 — ac2c02215_si_002.pdf [file ac2c02215_si_002.pdf]

Supporting information for:

Charting the proteoform landscape of the serum proteins by high-resolution native mass spectrometry

Dario A. T. Cramer[1,2], Vojtech Franc[1,2], Tomislav Caval[1,2,3], Albert J. R. Heck[1,2 *].

[1]Biomolecular Mass Spectrometry and Proteomics, Bijvoet Center for Biomolecular Research and Utrecht Institute for Pharmaceutical Science, University of Utrecht, Padualaan 8, Utrecht, 3584 CH, The Netherlands

[2]Netherlands Proteomics Centre, University of Utrecht, Padualaan 8, Utrecht, 3584 CH, The Netherlands

[3]current address, Stanford

Corresponding Author

[*]e-mail: a.j.r.heck@uu.nl, tel: +31 - 302536797

This supporting information contains:

Supplementary table and supplements legend 2

Materials and methods 3

Supplementary figure 1: Typical tandem mixed cation-anion exchange chromatogram. 5

Supplementary figure 2: Technical replicates of IEX on serum from donor 4. 5

Supplementary figure 3: Changes in the IEX chromatogram after the application of a 3-in-1 protein depletion. 6

Supplementary figure 4: Charge state deconvolution of A1AT. 7

Supplementary figure 5: Charge state deconvolution of CER. 8

Supplementary figure 6: Charge state deconvolution of HPX. 8

Supplementary figure 7: Native ESI-MS spectra of commercial serum A1AT, before and after enzymatic treatment. 9

Supplementary figure 8: Validation of high-mannose N-glycosylation on C3 and C3c using sialidase treatment. 10

Supplementary figure 9: Annotation and identification of proteoforma of complement C3 isolated from donor 2. 11

Supplementary figure 10: Mass spectra of C3 isolated from donor 2 after treatment with PNGase F. 12

Supplementary figure 11: MS/MS spectra and annotation of glycopeptides observed in the proteolystic digest on C3 purified from donor 1 and 2. 13

Supplementary figure 12: Native MS and annotation of proteoform profiles of HPX, following treatment with sialidase and PNGase F. 14

Supplementary figure 13: Native MS and annotation of hemopexin proteoform profiles following treatment with sialidase and PNGase F after charge deconvolution. 15

Supplementary figure 14: Native MS and annotation of CER following treatment with sialidase and PNGase F. 16

Supplementary figure 15: Incubation of holo-CER in 50 mM ammonium bicarbonate leads to the removal of a cluster of metal ions. 17

Supplementary figure 16: Deconvoluted native MS spectra of deglycosylated CER following incubation in PBS and AMBIC. 18

References 18

Supplementary table and supplements legend

Suppl. table 1: overview of donors and their physiological condition

Suppl. table 2: overview of proteins observed during IEX chromatography followed by native-MS analysis

Suppl. table 3: annotation of proteoforms typically observed when analyzing serum A1AT

Suppl. table 4: annotation of proteoforms observed in C3

Suppl. table 5: annotation of proteoforms observed in asialo-hemopexin

Suppl. table 6: annotation of proteoforms observed in asialo-ceruloplasmin

Supplemental file S1: native MS spectra of ceruloplasmin measured in all donors

Supplemental file S2: native MS spectra, raw and deconvoluted, of all proteins observed in the study

Materials and methods

Origin of serum samples

Individual serum samples from six healthy donors were provided by A. Meijer from Sanquin Research (Amsterdam, The Netherlands). Serum samples were obtained in accordance with the ethics board of Sanquin and after informed consent from the donors. Serum was separated from collected whole blood by centrifugation at 1800 x g for 20 minutes, transferred to 1.5 mL Eppendorf tubes, snap-frozen in liquid nitrogen and stored at -80 °C. Individual serum samples from four late-stage hepatocellular carcinoma patients and six late-stage pancreatic cancer patients were purchased from Discovery Life Sciences (Columbus, OH, USA). Further details on the serum samples are provided in Supplementary Table 1.

Chemicals and materials

As reference standards the human proteins A1AT, CER, and HPX were purchased from Sigma Aldrich (St. Louis, MO, USA). All chemicals and enzymes used, i.e., ammonium acetate (AMAC), tris(2-carboxyethyl)phosphine (TCEP), neuraminidase (sialidase) from *Arthrobacter ureafaciens* and peptide-N-glycosidase F (PNGase F) from *Elizabethkingia meningoseptica* were also purchased from Sigma Aldrich (St. Louis, MO, USA). The reference proteins A1AT, CER and HPX originated from pooled human plasma. Complement C3 isolated from pooled human plasma was acquired from Complementech (Tyler, TX, USA).

Depletion of three abundant serum proteins

Aliquots of 150 µL serum from each donor were filtered using a 0.22 µm filter (WAT200516 Acrodisc, USA) on a 1 mL syringe. To remove IgG, serotransferrin and albumin, aliquots were loaded on a 3-in-1 depletion column (HD-0301-10GFC, Good Biotech Corp., Taiwan) following the manufacturer's instructions. This process was automated using an elution robot (Favonian, Apeldoorn, The Netherlands). In brief, serum aliquots were loaded on the column and incubated with 1 mL PBS for 10 min, followed by six elution steps of 1 mL PBS (pH 7.5). Unwanted proteins were washed off using 40 mL 0.1 M glycine solution (pH 2.0). Each depleted serum sample was concentrated to approximately 150 µL using Millipore 4 mL (UFC801096, Merck Millipore, Ireland) 10 kDa MWCO filter spin tubes at 4,000 rcf for 10 minutes twice.

Fractionation of Serum Proteins

Aliquots of 150 µL serum from each donor were used. These serum samples, depleted for 3 proteins, were fractionated over an ion-exchange setup comprised of a tandem of cationic and anionic columns (PolyCAT A 204CT0510 and PolyWAX LP 204WX0510, PolyLC, USA) following a previously described method[1]. The columns were equilibrated with 100 µg of bovine serum albumin (BSA). One fraction was collected every 0.5 minutes (400 µL, 13-27 min). Elution of A1AT, CER, HPX and C3 was validated by comparing the retention times of commercial standards and their MS spectra. Retention times of all proteins are provided in Supplementary Table 2. A typical IEX chromatogram is shown in Supplementary Figure 1. The reproducibility of the IEX method was validated with a technical triplicate (Supplementary Figure 2).

Native MS Sample preparation

Protein fractions were buffer exchanged into 150 mM AMAC (pH 7.5) by ultrafiltration with a 10 kDa cut-off filter. Sialidase was used to remove sialic acid residues from several studied proteins and PNGase F was used to cleave off N-glycans. Deglycosylation was performed in PBS or 50 mM ammonium bicarbonate (AMBIC). Enzyme-treated samples were buffer exchanged prior to native MS analysis.

Native MS Analysis

Protein samples were analyzed on a modified Exactive Plus Orbitrap instrument with an extended mass range (Thermo Fisher Scientific, Bremen) as previously described[2] using a m/z range of 500-15,000. Voltage offsets on the transport multi-poles and ion lenses were tuned to achieve optimal transmission of the protein ions. Nitrogen was used in the higher-energy collisional dissociation (HCD) cell at a gas pressure of 6-8 x 10^{-10} bar. Spray voltage was set to 1.3 kV. Source fragmentation and collision energy were optimized for the analysis of each protein. Source temperature was set to

250 °C. Acquisition time per transient was set to obtain a resolution (at m/z 200) of 30,000. The instrument was mass calibrated using a solution of CsI.

Native MS Data Analysis

Accurate masses of proteoforms were extracted by deconvolution of the raw native MS spectra to zero-charge spectra using PMi Intact Mass software (ProteinMetrics, version 4.1-4.3). Resulting data were manually cross-checked with raw spectra. Analysis of PTM composition after deconvolution was done manually. Glycan structures and other PTMs were deduced based on reported glycopeptide data and removal of sialic acids and glycans. Cysteinylation was confirmed by reduction with TCEP. For calculations, average masses were used, namely hexose/mannose/galactose (Hex/Man/Gal, 162.1424 Da), N-Acetylglucosamine (NAc, 291.26 Da), fucose (Fuc, 146.14 Da), neuraminic acid (NeuAc, 291.26 Da) and cysteinylation (Cys, 121.16 Da). Symbols and text nomenclature are based on the recommendations of the Consortium for Functional Glycomics. Quantification of CER proteoforms for comparison between donors was done using relative intensities. Differences in glycosylation of CER in healthy and diseased individuals were measured by dividing the relative intensities of the proteoforms of 3-N-ceruloplasmin by 4-N-ceruloplasmin (number of N-glycosylation sites occupied). The difference between groups was statistically tested using Welch's t-test. All statistics were performed in Graphpad Prism software (version 9.0.0)

Peptide-centric LC-MS/MS analysis

C3 protein samples were digested in solution by adding 5 µg in 200 mM TRIS and reducing with 50 mM TCEP for 30 min at 60 °C. After cooling to RT, samples were alkylated with 200 mM CAA. This reaction was quenched with 50 mM TCEP and sodium deoxycholate (SDC) was added to 1%. Protein samples were digested for 4h using trypsin at an enzyme-to-protein ratio of 1:100, then overnight with Glu-C enzyme at a ratio of 1:75, at 37 °C. All digests were desalted using a protocol as previously described[3] using OASIS plates (Waters, Wexford, Ireland), dried and dissolved in 2% FA prior to LC-MS analysis.

Peptide-centric LC-MS/MS data analysis

Peptides of protein samples (100 ng) were separated and analyzed using an Ultimate HPLC nanoflow system coupled to an Exploris Orbitrap mass spectrometer (both Thermo Fisher Scientific, Bremen, Germany) as previously described[3]. In short, analysis was performed in positive ion mode using electrospray ionization. MS1 scans were obtained in a mass range from *m/z* 375 to 1600 at a resolution of 60,000. Data-dependent MS2 acquisition was at a resolution of 15,000 with a mass range from *m/z* 40 to 2500. Raw data were searched manually to identify glycan fragment ions and confirm results obtained by data interpretation with Byonic software (v4.3.4, Protein Metrics Inc., San Fransisco, USA). Modifications included in the search were human N- and O-glycans, Glu- and Gly-pyrolysis, Met- and Try-oxidation.

UV-VIS absorption of apo- and holo-ceruloplasmin

Protein samples were buffer exchanged and resuspended into a 1 µg/µL solution in PBS or 50 mM AMBIC. On a 96 well-plate, 200 µL of each sample was loaded and the respective buffers as baseline controls. Absorption was measured using a Spectrostar Nano (BMG Labtech, Ortenberg, Germany) from wavelengths 900 to 210 nm at intervals of 2 nm. If required, samples were incubated with PNGase for 48h at 37 °C. The acquired data was processed manually.

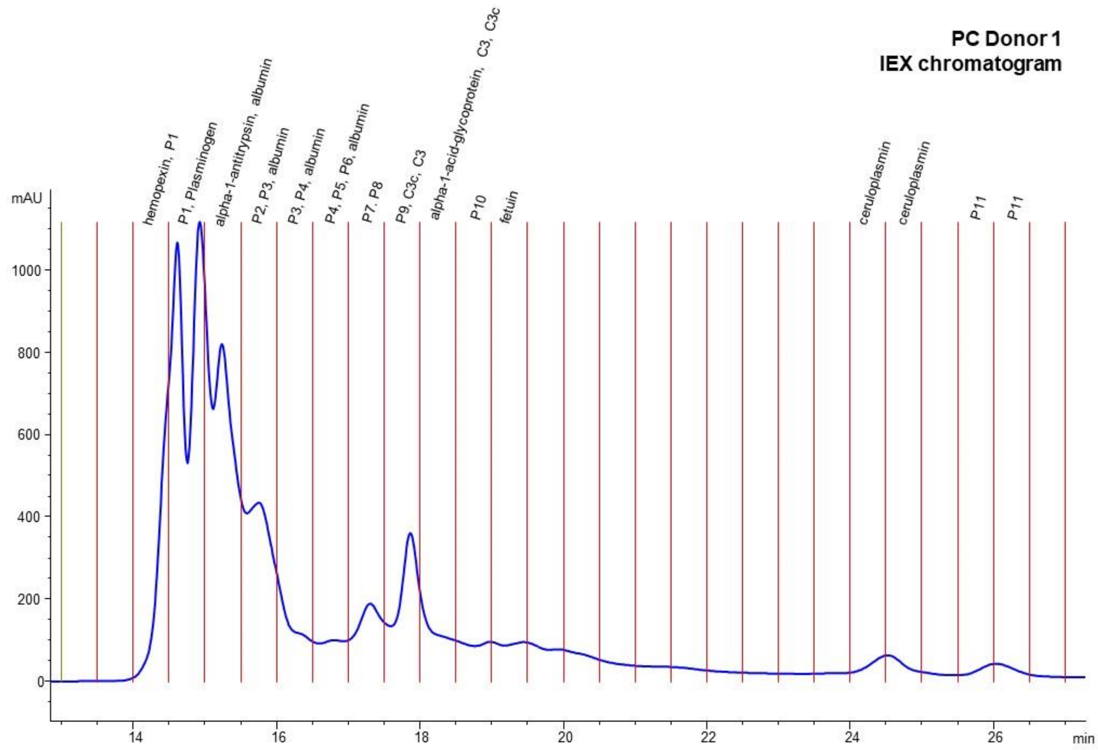

Supplementary figure 1: Typical tandem mixed cation-anion exchange chromatogram. The detected proteins are marked over the fractions in which they elute. Some overlap (co-elution) can be seen, such as for albumin or C3.

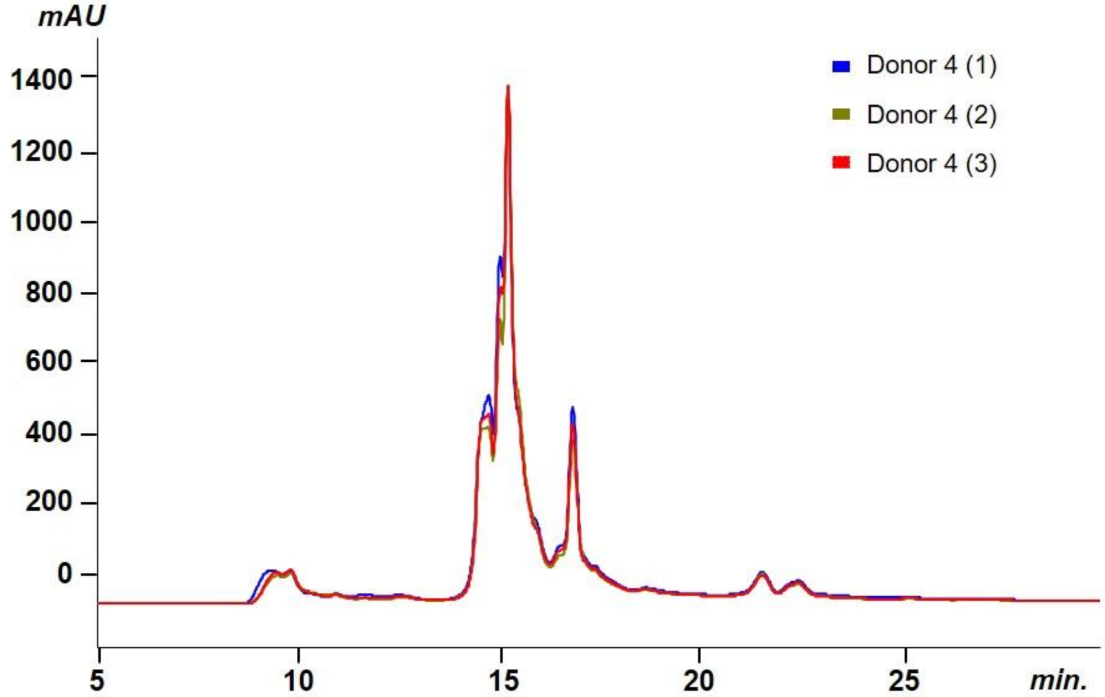

Supplementary figure 2: Technical replicates of IEX on serum from donor 4. Serum was depleted using the 3-in-1 protein depletion column, concentrated and ran over IEX on three separate occasions with the described method. No great change in retention times as well as peak height was observed over the three runs.

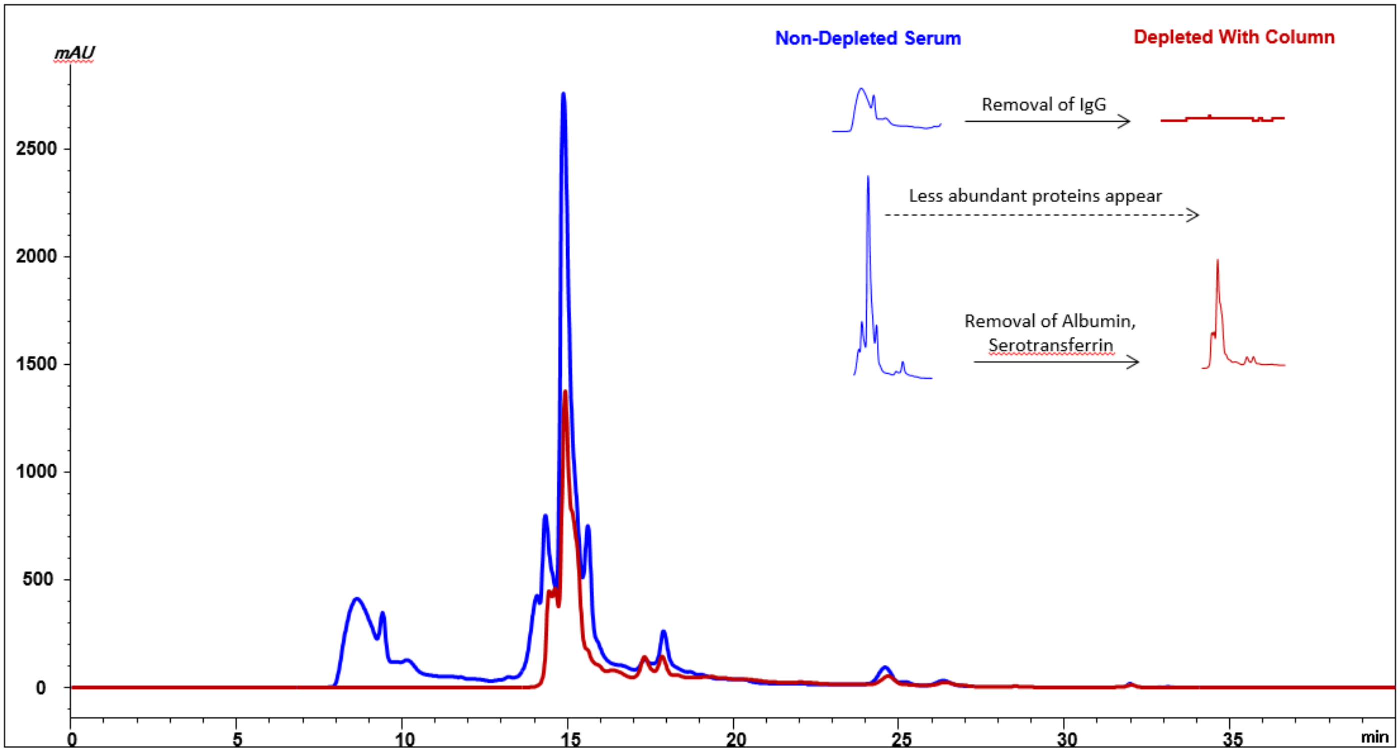

Supplementary figure 3: Changes in the IEX chromatogram after the application of a 3-in-1 protein depletion. Serum was analyzed both without and after being depleted using the 3-in-1 depletion column. The 3-in-1 affinity depletion column aims to remove albumin, IgG and serotransferrin. From MS analysis we learned that the main peak still contains albumin, albeit that this no longer overshadows other proteins after depletion. Serotransferrin was found coeluting in many fractions, likely due to its ability to bind different amounts of metal ions, but no longer detected after depletion. IgG was thought to mostly be contained in the broad peak from 8-10 minutes, possibly owing to its relatively higher PI compared to common serum proteins such as albumin, HPX, fetuin etc.

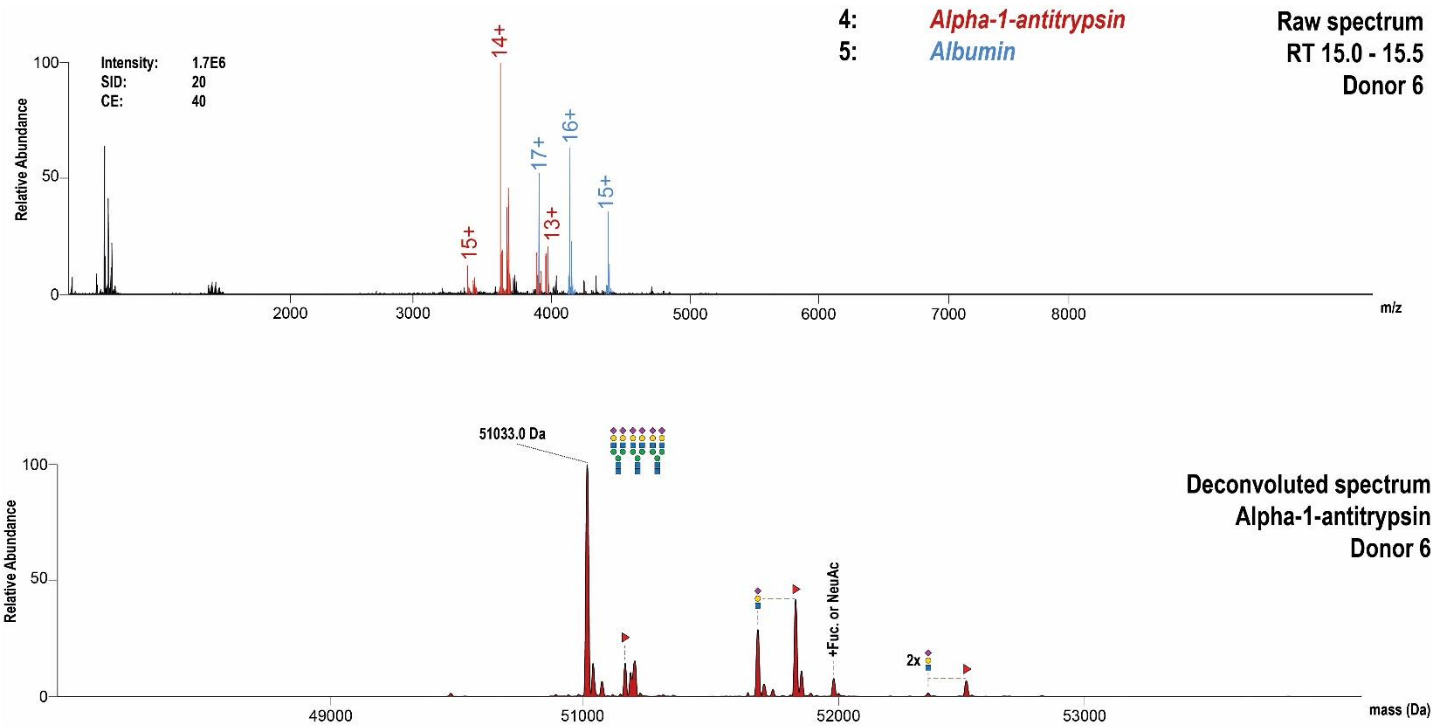

Supplementary figure 4: Charge state deconvolution of A1AT. Eluting in the main peak of the chromatogram around 15 minutes is A1AT (in red). The depletion of albumin as described in the method prior to IEX chromatography drastically reduces the abundance of albumin (in blue) which however still co-elutes with A1AT. Because of the depletion we are able to effectively deconvolute and annotate A1AT.

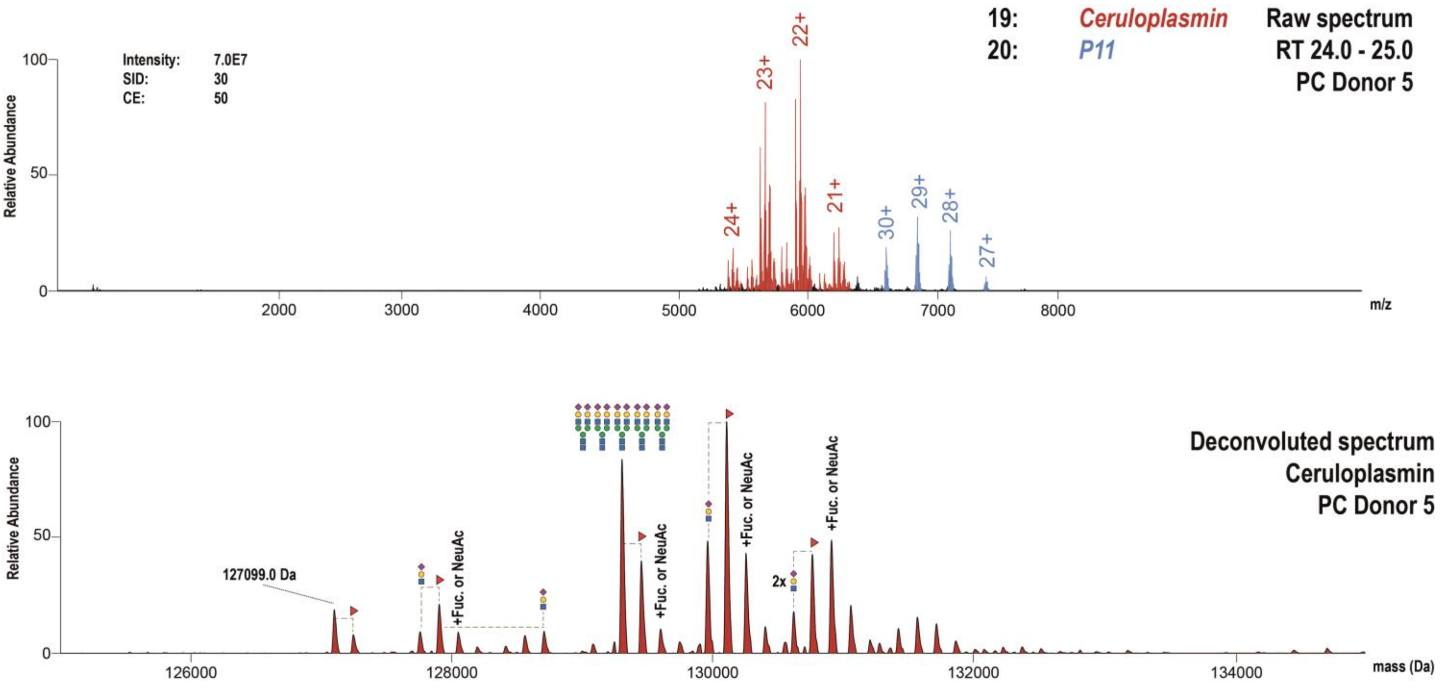

Supplementary figure 5: Charge state deconvolution of CER. CER (in red), eluting from 24 to 25 minutes can be properly deconvoluted despite co-eluting with protein P11 (25.5-26.5 minutes). Regardless of the relatively higher amount of glycoproteoforms and higher mass of CER, distinct proteoforms were deconvoluted and annotated.

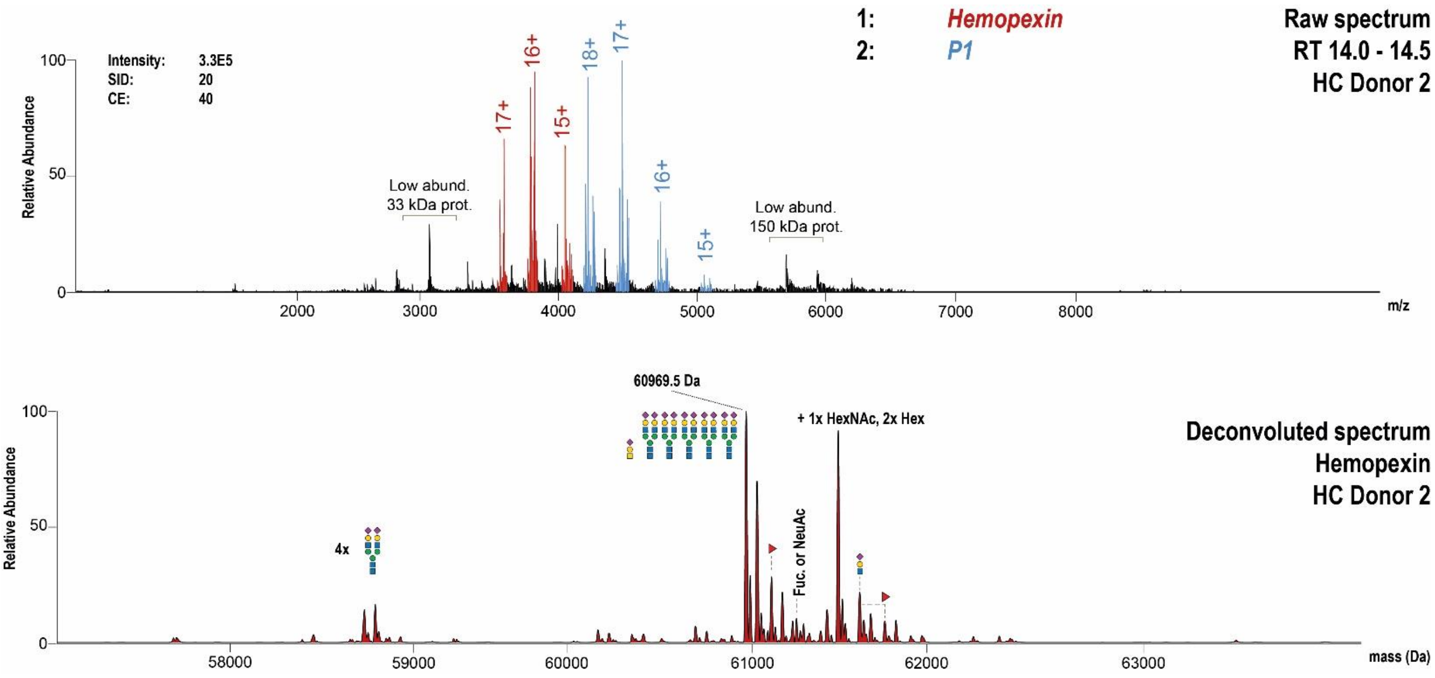

Supplementary figure 6: Charge state deconvolution of HPX. HPX (in red) elutes around 14 min. and, despite co-eluting with unidentified protein P1, the mass spectrum can be accurately deconvoluted and annotated due to the differences in mass.

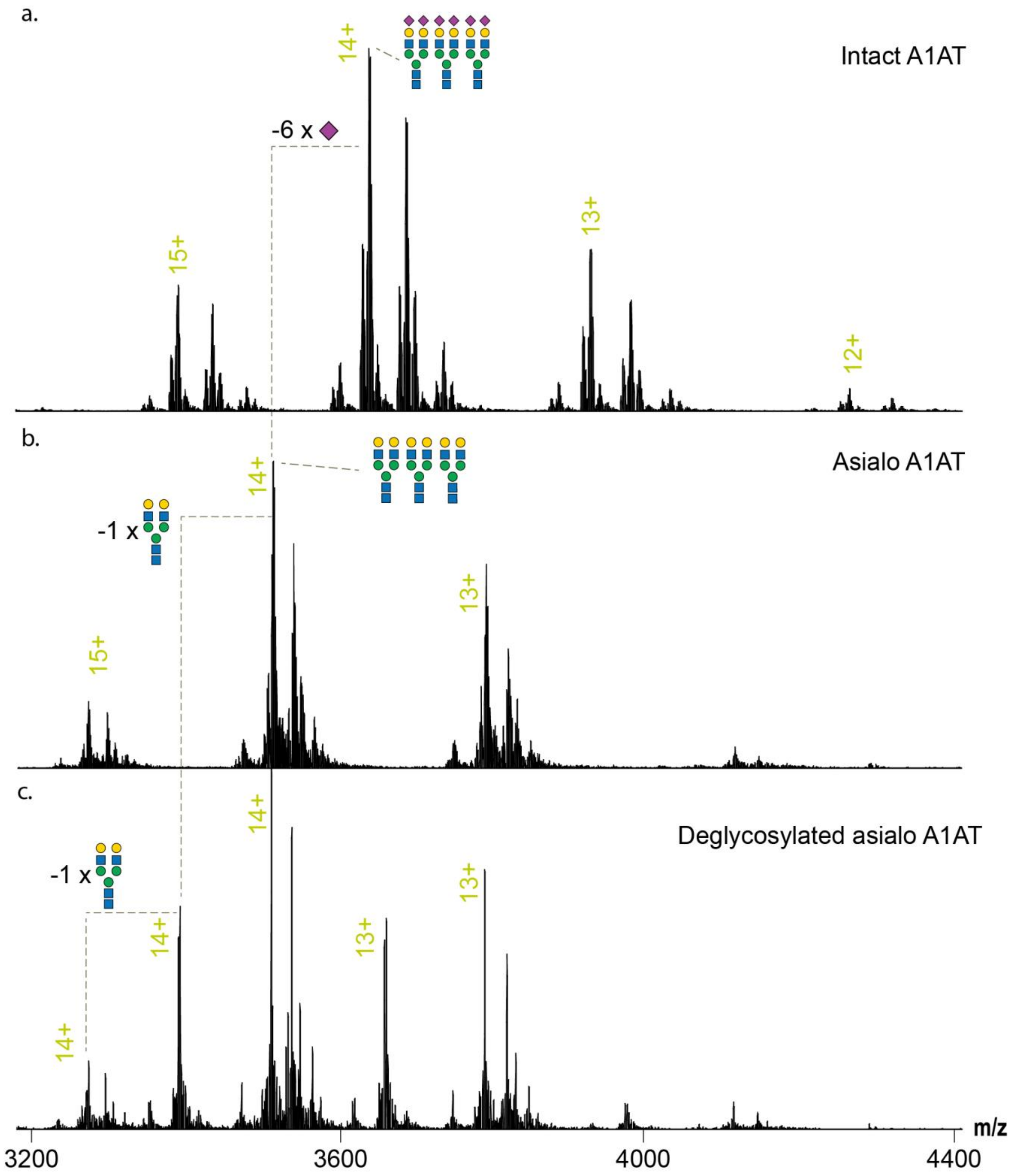

Supplementary figure 7: Native ESI-MS spectra of commercial serum A1AT, before and after enzymatic treatment. a. intact serum A1AT is observed with three complex N-glycans. **b**. Following treatment with a sialidase asialo A1AT displays the loss of 6 sialic acids. This spectrum allows us to annotate fucoses and branching without overlap with the mass of a sialic acid. **c**. Native MS spectrum of serum A1AT, following treatment with the sialidase and PNGaseF under native conditions. A1AT is partially deglycosylated with PNGase F, with the main species present still containing the N-glycans. The loss of up to two N-glycans is observed at an increasingly low abundance per loss of glycan.

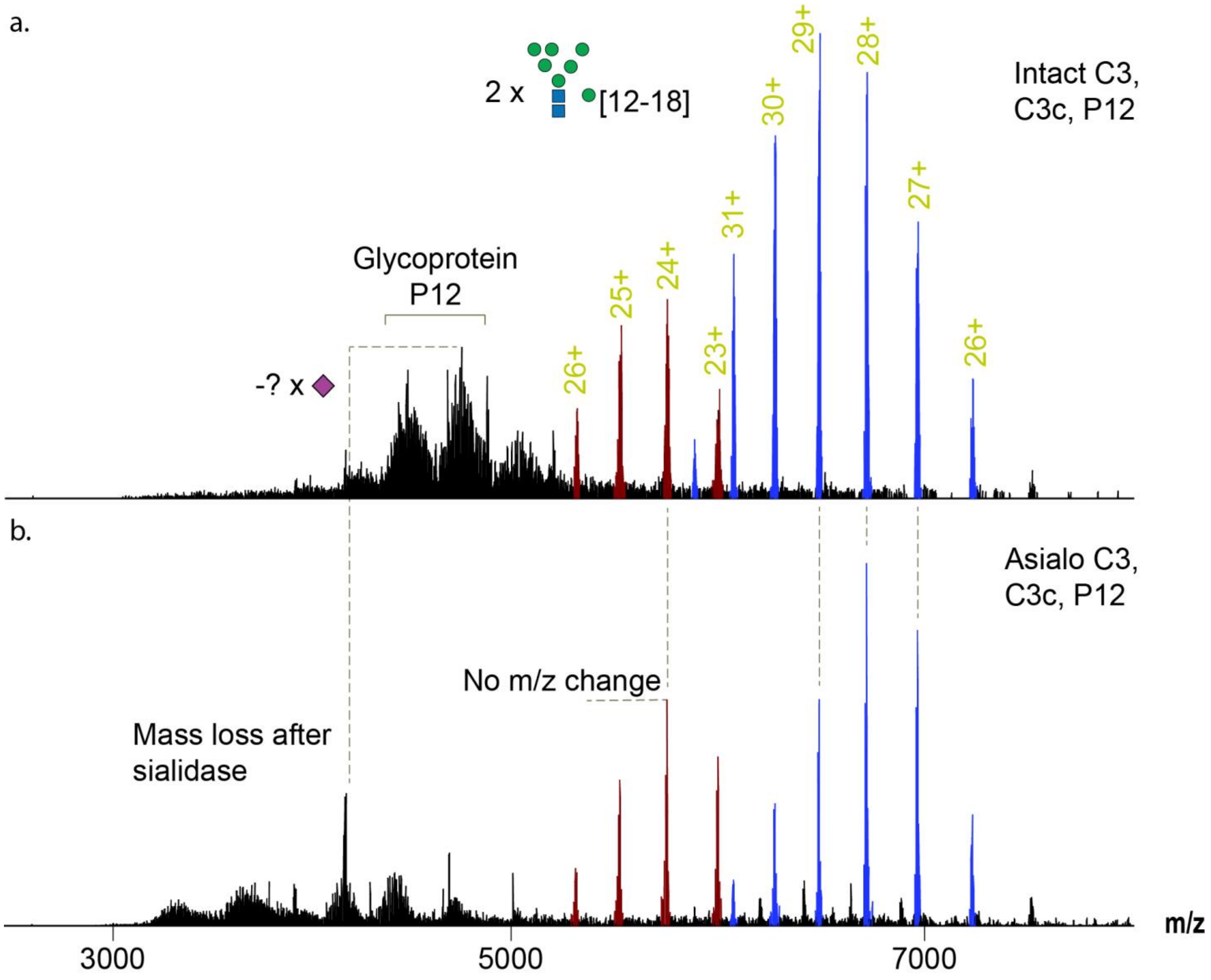

Supplementary figure 8: Validation of high-mannose N-glycosylation on C3 and C3c using sialidase treatment. a. Co-eluting at RT 17 min., native MS analysis shows a 187 kDa and a 137 kDa protein both carrying two high mannose N-glycans, identified to be C3 (blue) and C3c (red). Another protein coeluting with C3 and C3c is a highly decorated unidentified glycoprotein, termed P12. **b**. Treatment with sialidase shows a substantial mass shift of an considerable amount of sialic acids for the glycoprotein P12, but no apparent mass shift for C3 or C3c confirming the glycans on C3 and C3c to not be sialylated. Samples were obtained from PC donor 6.

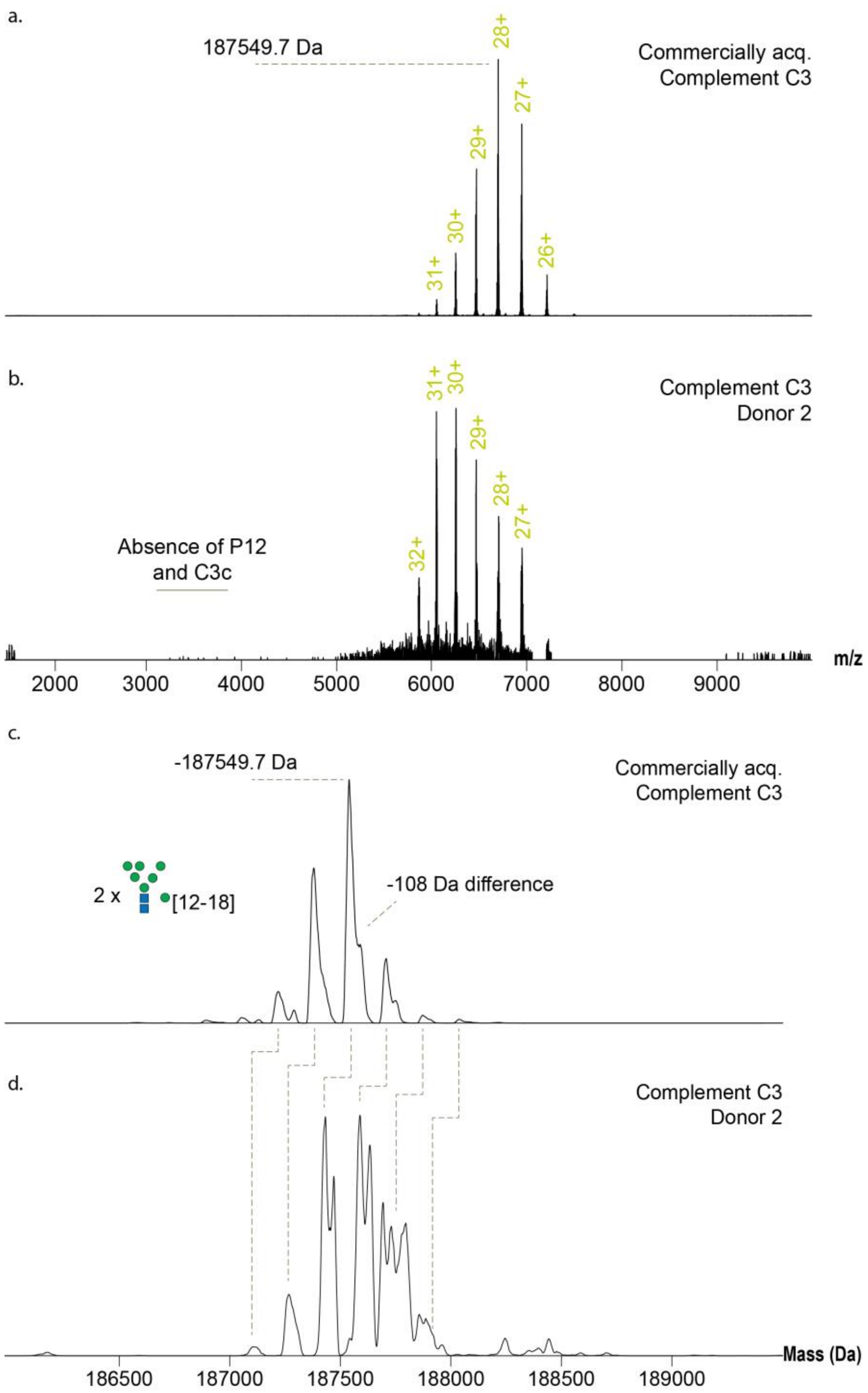

Supplementary figure 9: Annotation and identification of proteoforma of complement C3 isolated from donor 2. a. The native mass spectrum of commercially available C3 (Complement technologies) shows a relatively simple charge state distribution and proteoform profile. **b**. C3 isolated from donor 2 is observed at a similar charge state distribution **c**. Deconvoluted mass spectrum of commercial C3. The measured masses can be explained by the protein backbone mass of C3 (subtracting 2 Da per disulfide bond), but only when we assume that C3 carries two oligomannose glycans containing up to 18 mannoses. The mass differences in between the four dominant peaks are close to 162 Da (i.e. additional mannose). **d**. Deconvoluted mass spectrum of C3 of donor 2 reveals a rather similar spectrum albeit with an overall mass difference of approximately 100 Da is observed. This speculatively can be attributed to the frequently observed Arg102Gly allotype variant dominantly present in this donor.

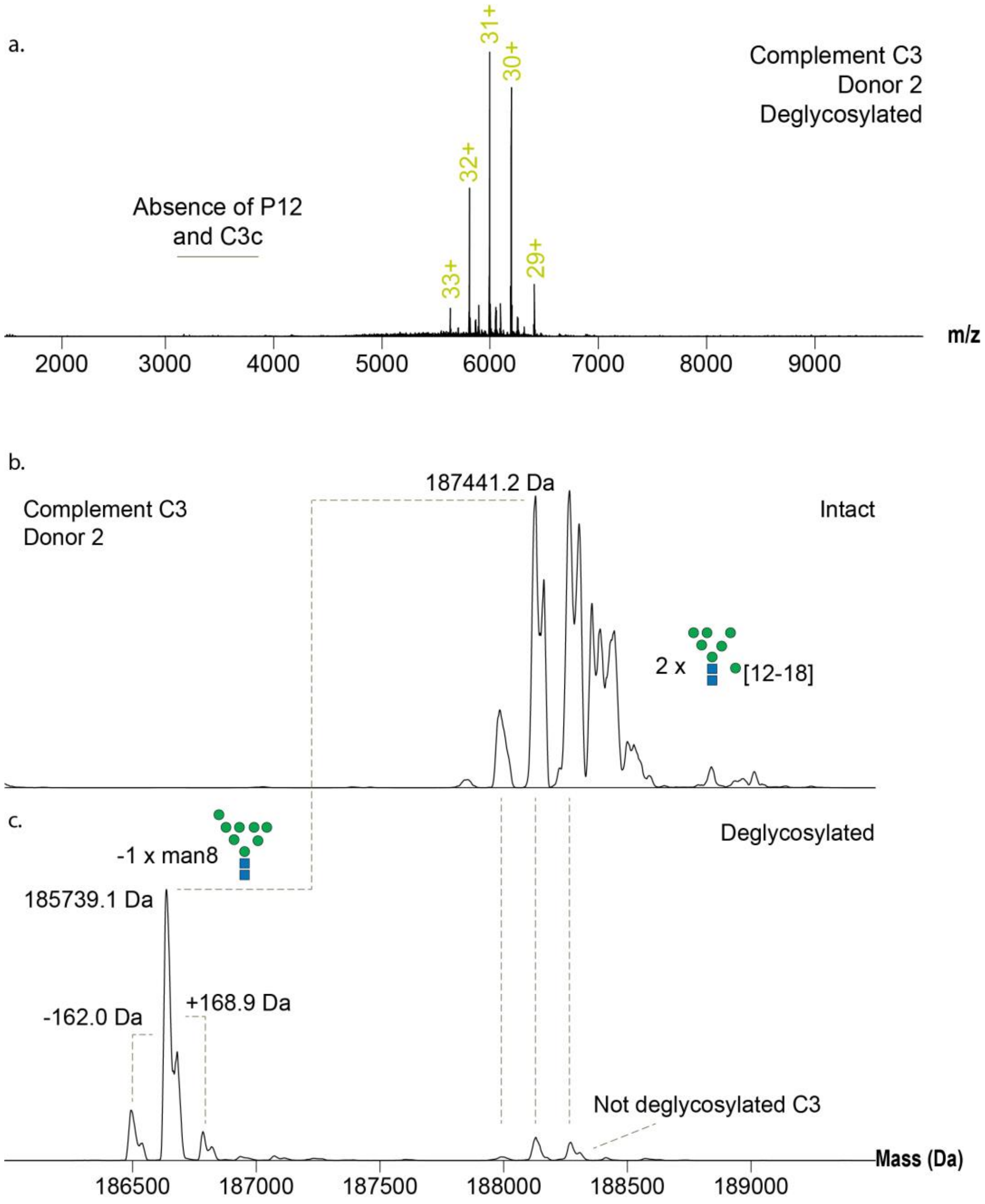

Supplementary figure 10: Mass spectra of C3 isolated from donor 2 after treatment with PNGase F. a. The raw spectrum of C3 from donor 2 after deglycosylation shows a cleaner spectrum (see supplementary figure 10). **b**. The untreated intact C3 from donor 2 for comparison, as shown also in supplementary figure 10. **c**. After deglycosylation, specifically one oligomannose glycan is removed, and one if likely more resistant to cleavage. The most dominant signal corresponds to the removal of a man8 glycan. Still, after deglycosylation, mass shifts from hexose additions (mannoses) are observed. A small amount of C3 is not deglycosylated at all after treatment with PNGase F.

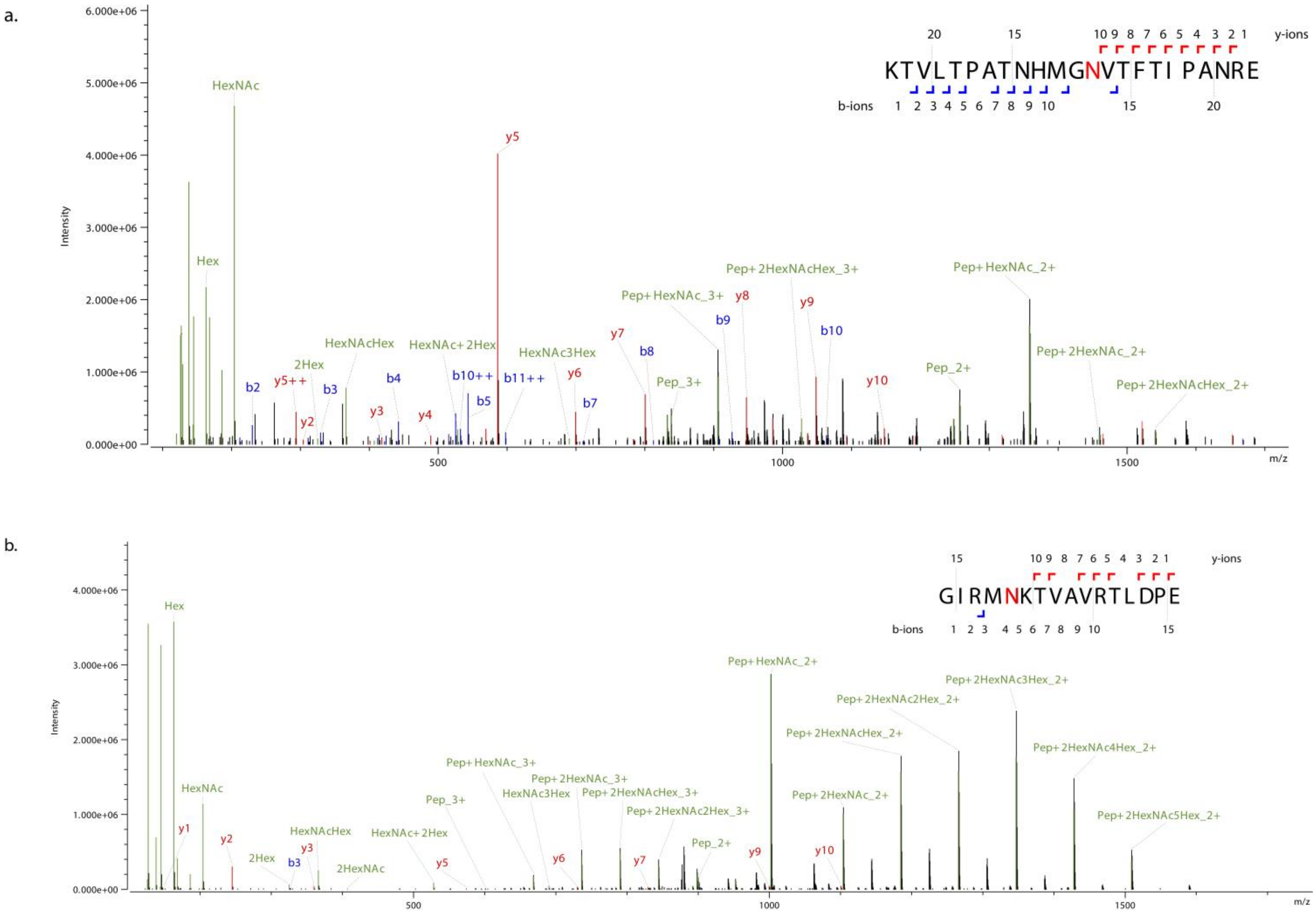

Supplementary figure 11: MS/MS spectra and annotation of glycopeptides observed in the proteolystic digest on C3 purified from donor 1 and 2. a. MS/MS spectrum (HCD) of a 23 amino acid long glycopeptide shows the b and y ions annotated as well as glycan fragments and the unfragmented peptide with partially fragmented glycans. The glycan type is identified to be an oligomannose glycan and is positioned on Asn 63. The peptide elutes at 20.5 min and had a peptide precursor mass of 3502.56 Da (Donor 2). **b**. MS/MS spectrum (HCD) of a 16 amino acid glycopeptide reveals another glycan on site Asn 917, which compared to the previous is even less fragmented at the peptide level, yet more so on the glycan level. This glycan is also identified to be an oligomannose glycan showing a range of 1-5 hexoses on 2 HexNAcs on the unfragmented peptide. This peptide elutes at 26.5 min and had a peptide precursor mass of 3890.79 Da (Donor 1). Both these N-glycosylation site have been previously annotated, as summarized in Nextprot (www.nextprot.org/entry/NX_P01024/sequence).

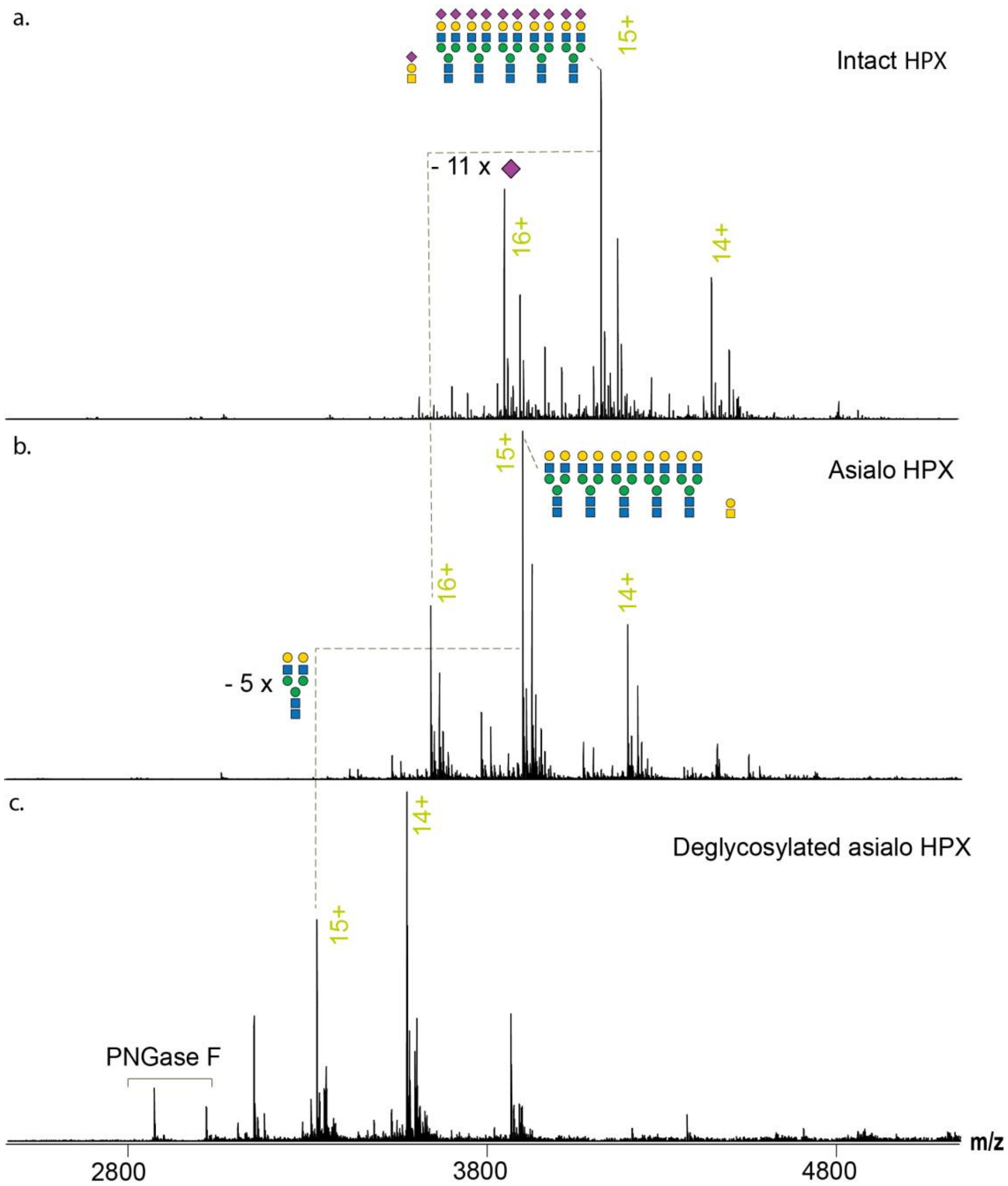

Supplementary figure 12: Native MS and annotation of proteoform profiles of HPX, following treatment with sialidase and PNGase F. a. Intact HPX can harbour four to five complex N-glycans and one O-glycan. Native MS spectrum obtained from a sample purified from donor 4. b. The removal of sialic acids with sialidase confirms the annotated amount of sialidases on these glycans and allows for the distinction between two fucoses and one sialic acid. c. After treatment with PNGase F, one O-glycan is still present on HPX but the five N-glycans are (mostly) enzymatically removed. Further glycan annotation is then possible by observing the mass differences between proteoforms.

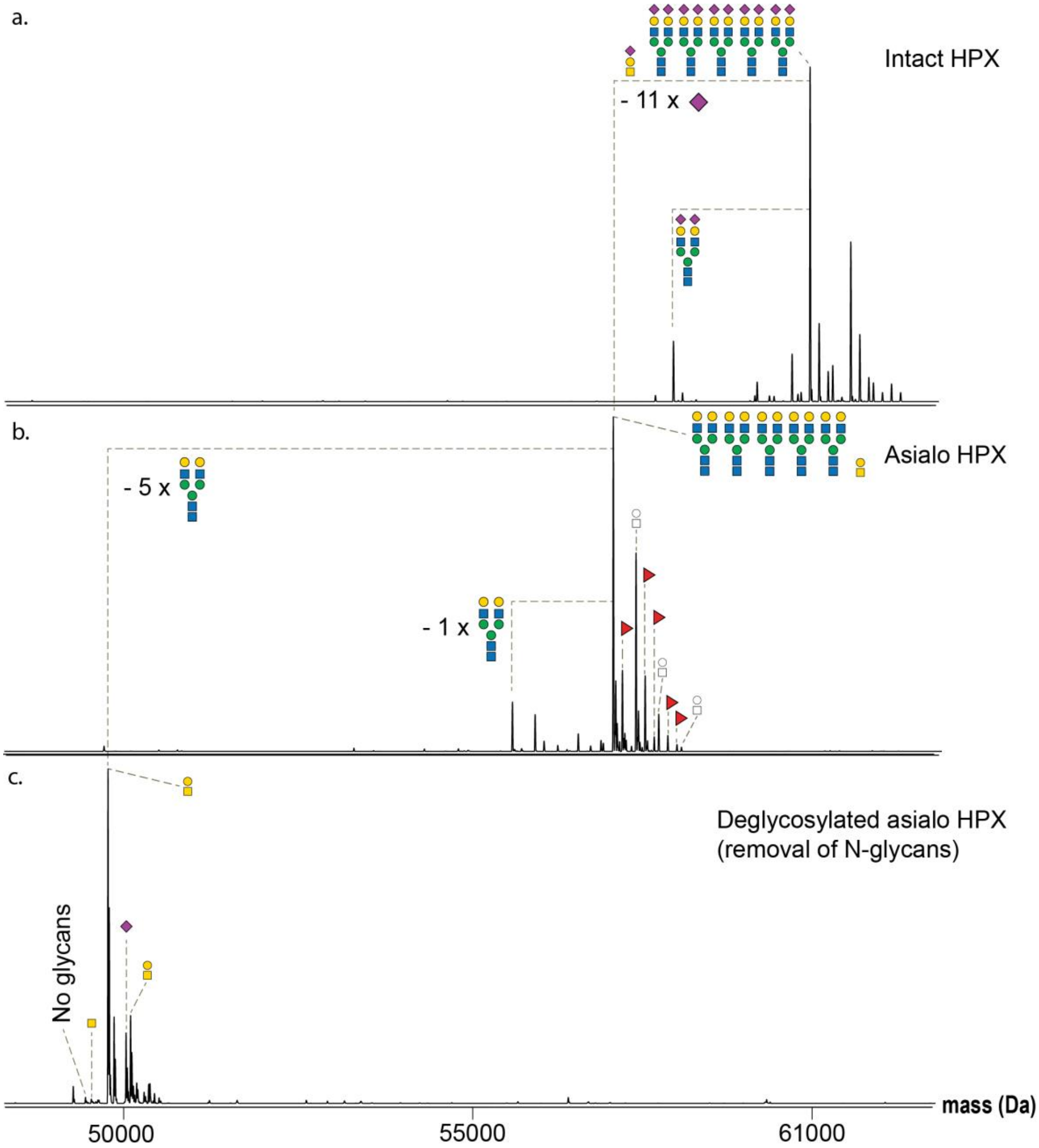

Supplementary figure 13: Native MS and annotation of hemopexin proteoform profiles following treatment with sialidase and PNGase F after charge deconvolution. a. Intact HPX has four to five complex N-glycans and one O-glycan. **b**. The removal of sialic acids with sialidase confirms the amount of sialidases on these glycans and allows us to confidently annotate fucoses and glycan branching. **c**. After treatment with PNGase F, one O-glycan is still present on HPX as well as one not fully removed sialic acid. Additionally, hemopexin seems to have a small amount of proteoforms with no O-glycosylation or the presence of only one GalNAc. All the shown data relate to Supplementary Figure 13.

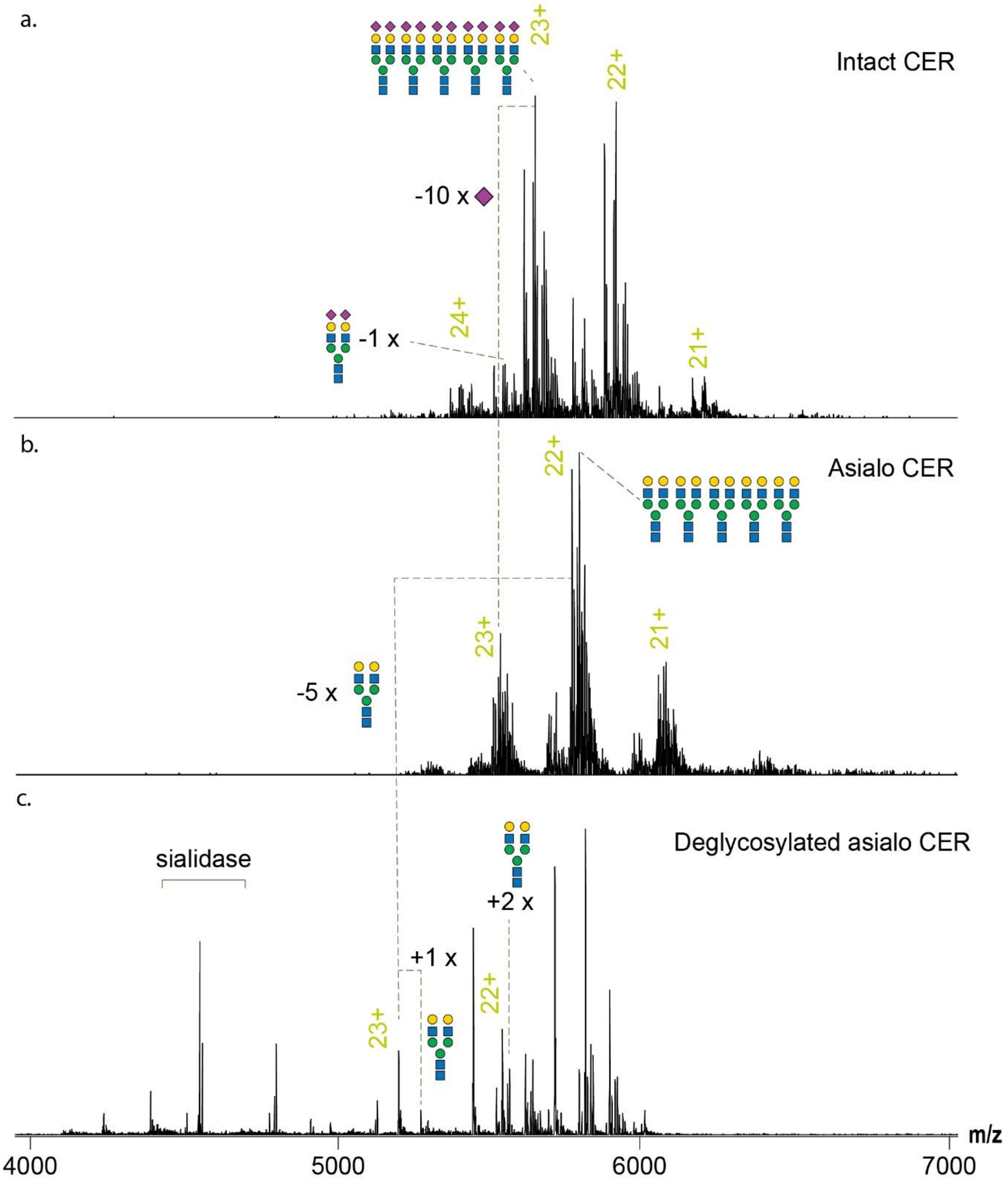

Supplementary figure 14: Native MS and annotation of CER following treatment with sialidase and PNGase F. a. Native MS spectrum of CER is shown with four complex N-glycans. **b**. Asialo CER is annotated after treatment with sialidase, to annotate fucoses and branching without overlap in mass of a sialic acid. It is shown that CER has either three or four biantennary complex N-glycans based on the released number of sialic acids. **c**. CER is either fully or partially deglycosylated with PNGase F, showing a single peak corresponding to its theoretical backbone mass, or the occupancy of one to two glycan sites.

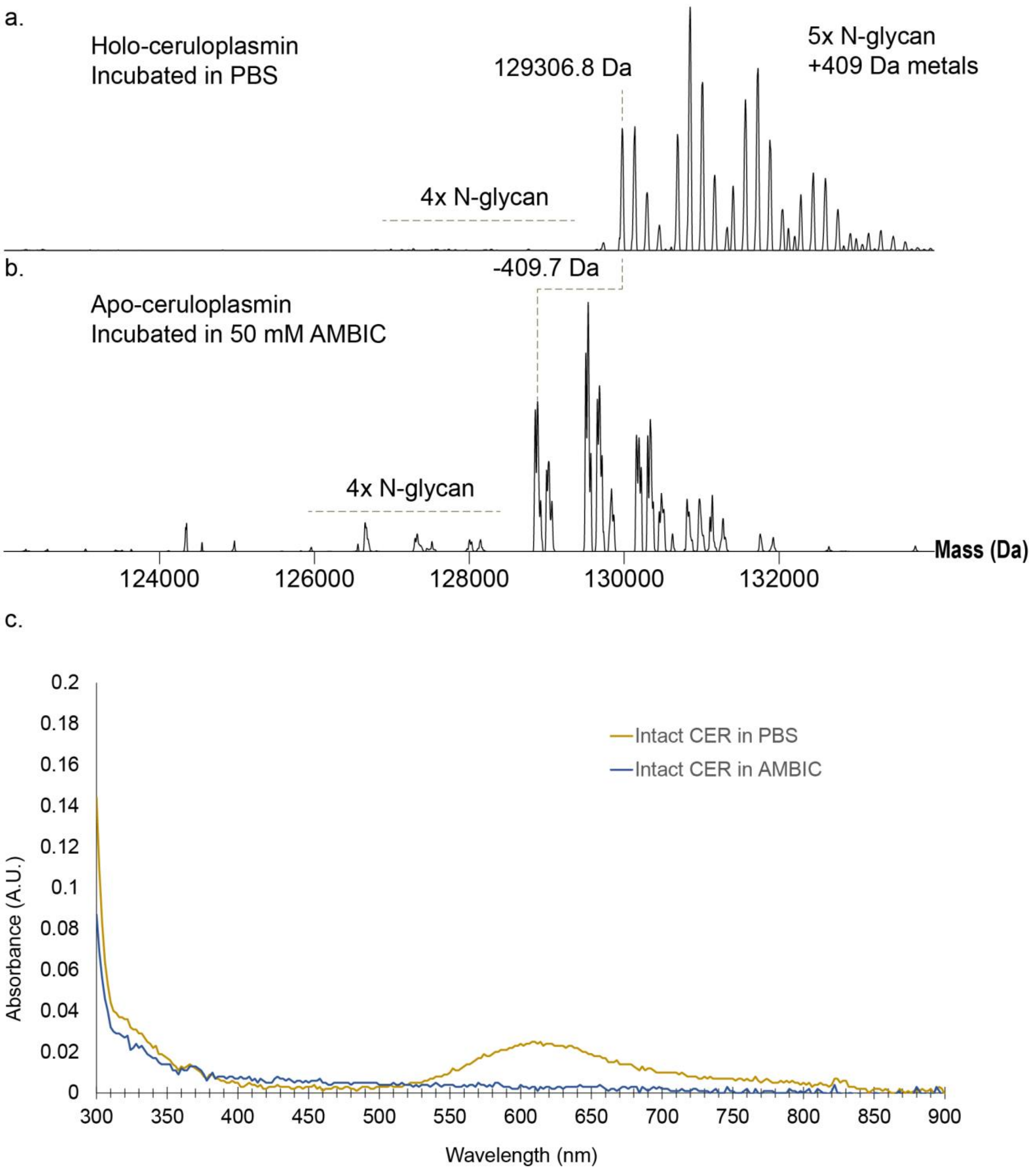

Supplementary figure 15: Incubation of holo-CER in 50 mM ammonium bicarbonate leads to the removal of a cluster of metal ions. a. native MS spectrum of CER with a range of proteoforms corresponding to changes in glycosylation, matching the mass of the peptide backbone sequence, the glycan masses leaving an unannotated mass addition of on average 409 Da. **b**. This 409 ±5 Da mass difference, which potentially be annotated to 6 Cu ions and 1 Ca ion, disappears after a 48h incubation in 50 mM ammonium bicarbonate, forming apo-CER. Each peak in 16b corresponds to a peak in 16a minus about 409 Da. It is well-known that holo-CER contains 6 tightly bound Cu ions in its structure. **c**. Intact CER in PBS shows some absorbance at 610 nm which can be attributed to a type I copper in the protein. In 50 mM AMBIC, five of six, or all coppers are removed. The absorbance spectra of intact CER in AMBIC thus also shows no absorbance at 610 nm.

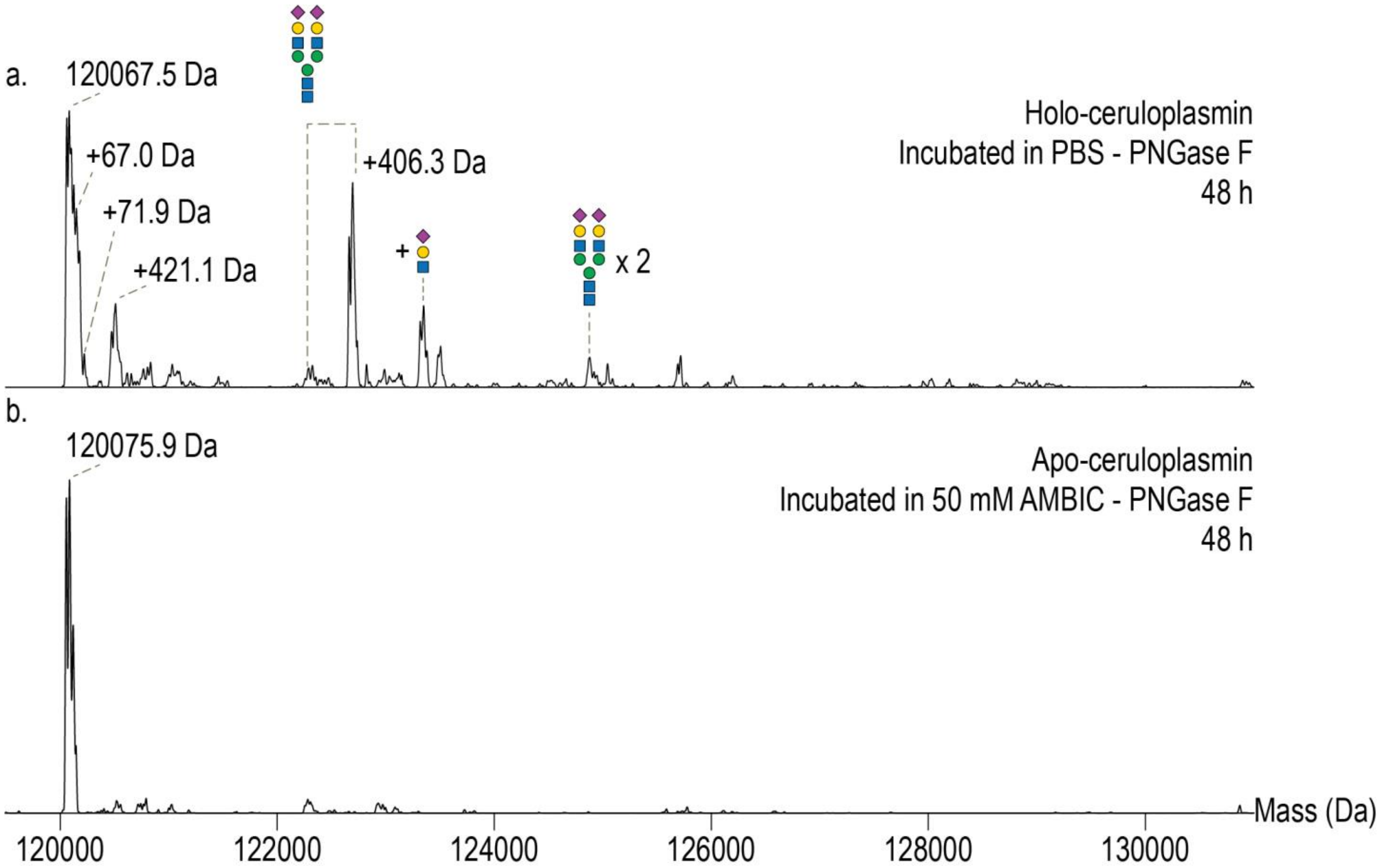

Supplementary figure 16: Deconvoluted native MS spectra of deglycosylated CER following incubation in PBS and AMBIC. a. 48h incubation of CER with PNGase F in PBS partially removes the glycans. The fully deglycosylated apo-CER has also lost the whole set of metal ions, but intriguingly the not-fully deglycosylated CER shows more dominant peaks that also contain all the metal ions (holo-CER). **b**. In contrast, 48h incubation with PNGase F in 50 mM AMBIC practically removes all glycans and leaves only apo-CER.

References

(1) Franc, V.; Zhu, J.; Heck, A. J. R. Comprehensive Proteoform Characterization of Plasma Complement Component C8αβγ by Hybrid Mass Spectrometry Approaches. *J. Am. Soc. Mass Spectrom.* **2018**, *29* (6), 1099–1110. https://doi.org/10.1007/s13361-018-1901-6.

(2) Rose, R. J.; Damoc, E.; Denisov, E.; Makarov, A.; Heck, A. J. R. High-Sensitivity Orbitrap Mass Analysis of Intact Macromolecular Assemblies. *Nat. Methods* **2012**, *9*, 1084.

(3) Luijkx, Y. M. C. A.; Henselijn, A. J.; Bosman, G. P.; Cramer, D. A. T.; Giesbers, K. C. A. P.; van 't Veld, E. M.; Boons, G.-J.; Heck, A. J. R.; Reiding, K. R.; Strijbis, K.; Wennekes, T. Detection of Bacterial α-l-Fucosidases with an Ortho-Quinone Methide-Based Probe and Mapping of the Probe-Protein Adducts. *Molecules* . 2022. https://doi.org/10.3390/molecules27051615.
