# Supplementary material for: Charting the Proteoform Landscape of Serum Proteins in Individual Donors by High-Resolution Native Mass Spectrometry
Source: Anal Chem. 2022 Sep 8;94(37):12732–41. doi: 10.1021/acs.analchem.2c02215 (PMC9494300; doi:10.1021/acs.analchem.2c02215)
Supplement: Supplementary file 3 — ac2c02215_si_003.pdf [file ac2c02215_si_003.pdf]

Supporting information for:

Charting the proteoform landscape of the serum proteins by high-resolution native mass spectrometry

Dario A. T. Cramer[1,2], Vojtech Franc[1,2], Tomislav Caval[1,2,3], Albert J. R. Heck[1,2 *].

[1]Biomolecular Mass Spectrometry and Proteomics, Bijvoet Center for Biomolecular Research and Utrecht Institute for Pharmaceutical Science, University of Utrecht, Padualaan 8, Utrecht, 3584 CH, The Netherlands

[2]Netherlands Proteomics Centre, University of Utrecht, Padualaan 8, Utrecht, 3584 CH, The Netherlands

[3]current address, Stanford

Corresponding Author

[*]e-mail: a.j.r.heck@uu.nl, tel: +31 - 302536797

This file contains:

Supplement S1

Native MS spectra of ceruloplasmin measured in all donors

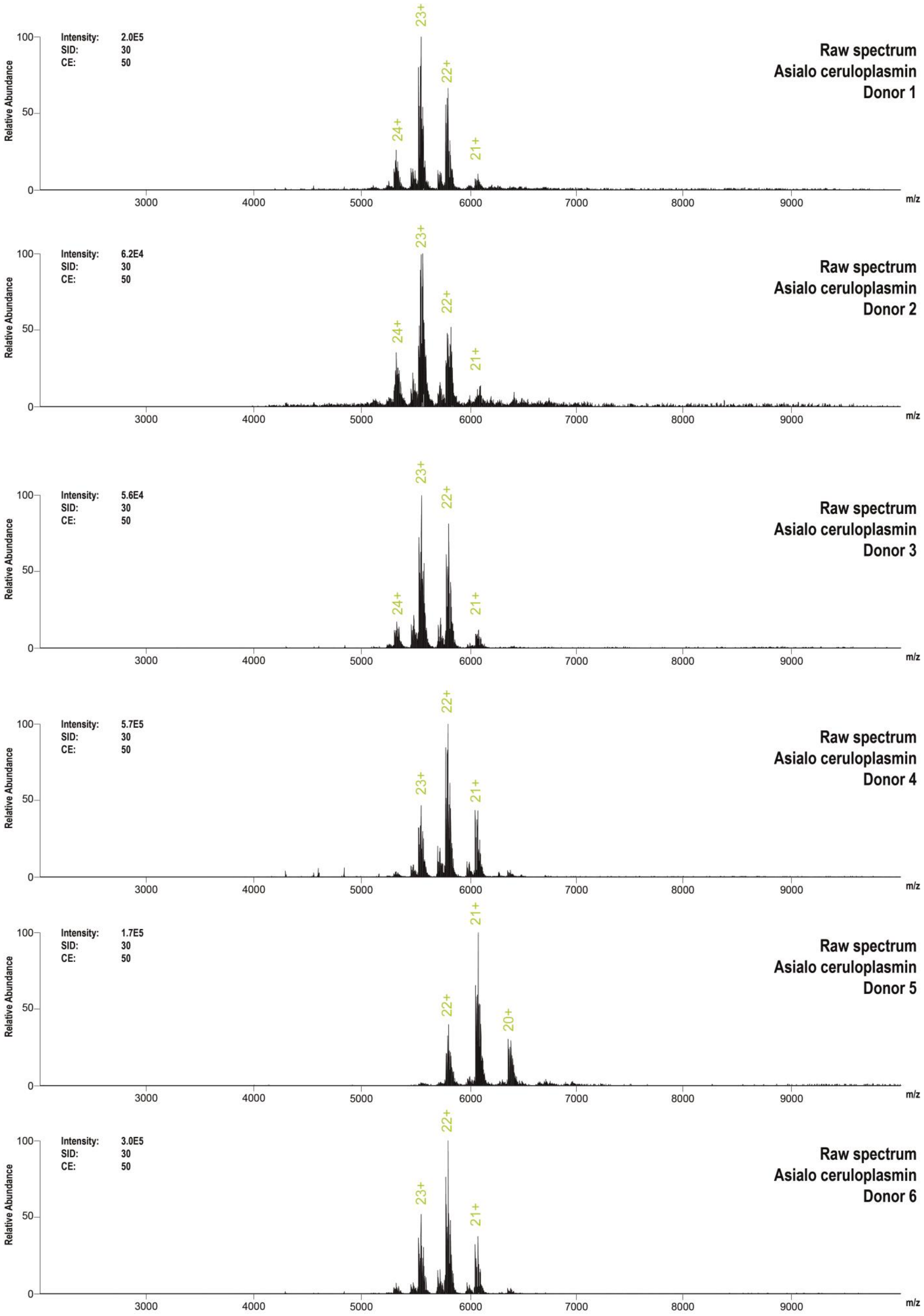
Intensity: 2.0E5
SID: 30
CE: 50
Raw spectrum
Asialo ceruloplasmin
Donor 1
24+
23+
22+
21+
Intensity: 6.2E4
SID: 30
CE: 50
Raw spectrum
Asialo ceruloplasmin
Donor 2
24+
23+
22+
21+
Intensity: 5.6E4
SID: 30
CE: 50
Raw spectrum
Asialo ceruloplasmin
Donor 3
24+
23+
22+
21+
Intensity: 5.7E5
SID: 30
CE: 50
Raw spectrum
Asialo ceruloplasmin
Donor 4
23+
22+
21+
Intensity: 1.7E5
SID: 30
CE: 50
Raw spectrum
Asialo ceruloplasmin
Donor 5
22+
21+
20+
Intensity: 3.0E5
SID: 30
CE: 50
Raw spectrum
Asialo ceruloplasmin
Donor 6
23+
22+
21+
Relative Abundance
0
50
100
3000
4000
5000
6000
7000
8000
9000
m/z

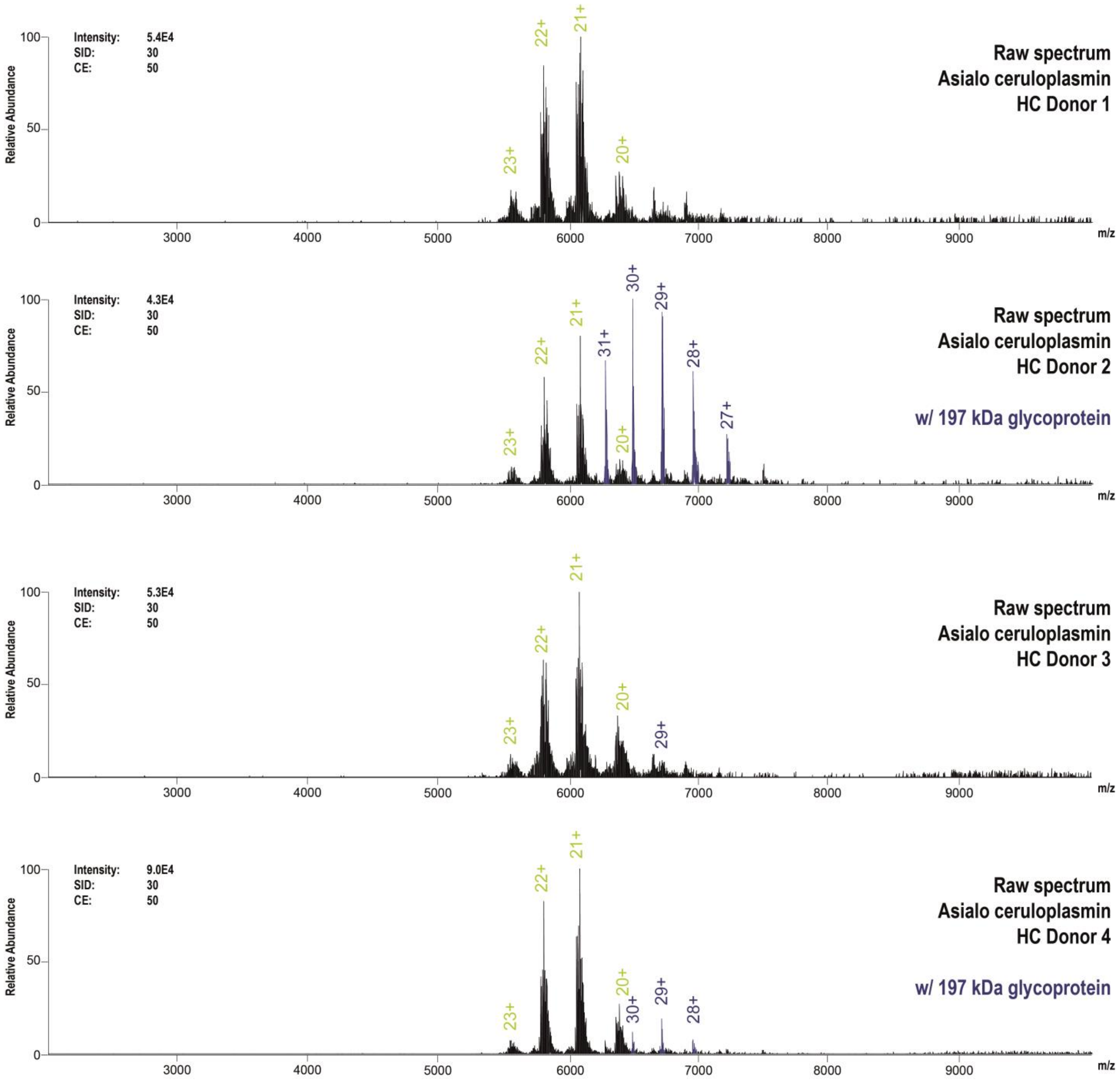
Intensity: 5.4E4
SID: 30
CE: 50
Raw spectrum
Asialo ceruloplasmin
HC Donor 1
23+
22+
21+
20+
Relative Abundance
m/z
Intensity: 4.3E4
SID: 30
CE: 50
Raw spectrum
Asialo ceruloplasmin
HC Donor 2
w/ 197 kDa glycoprotein
23+
22+
21+
31+
30+
20+
29+
28+
27+
Intensity: 5.3E4
SID: 30
CE: 50
Raw spectrum
Asialo ceruloplasmin
HC Donor 3
23+
22+
21+
20+
29+
Intensity: 9.0E4
SID: 30
CE: 50
Raw spectrum
Asialo ceruloplasmin
HC Donor 4
w/ 197 kDa glycoprotein
23+
22+
21+
20+
30+
29+
28+

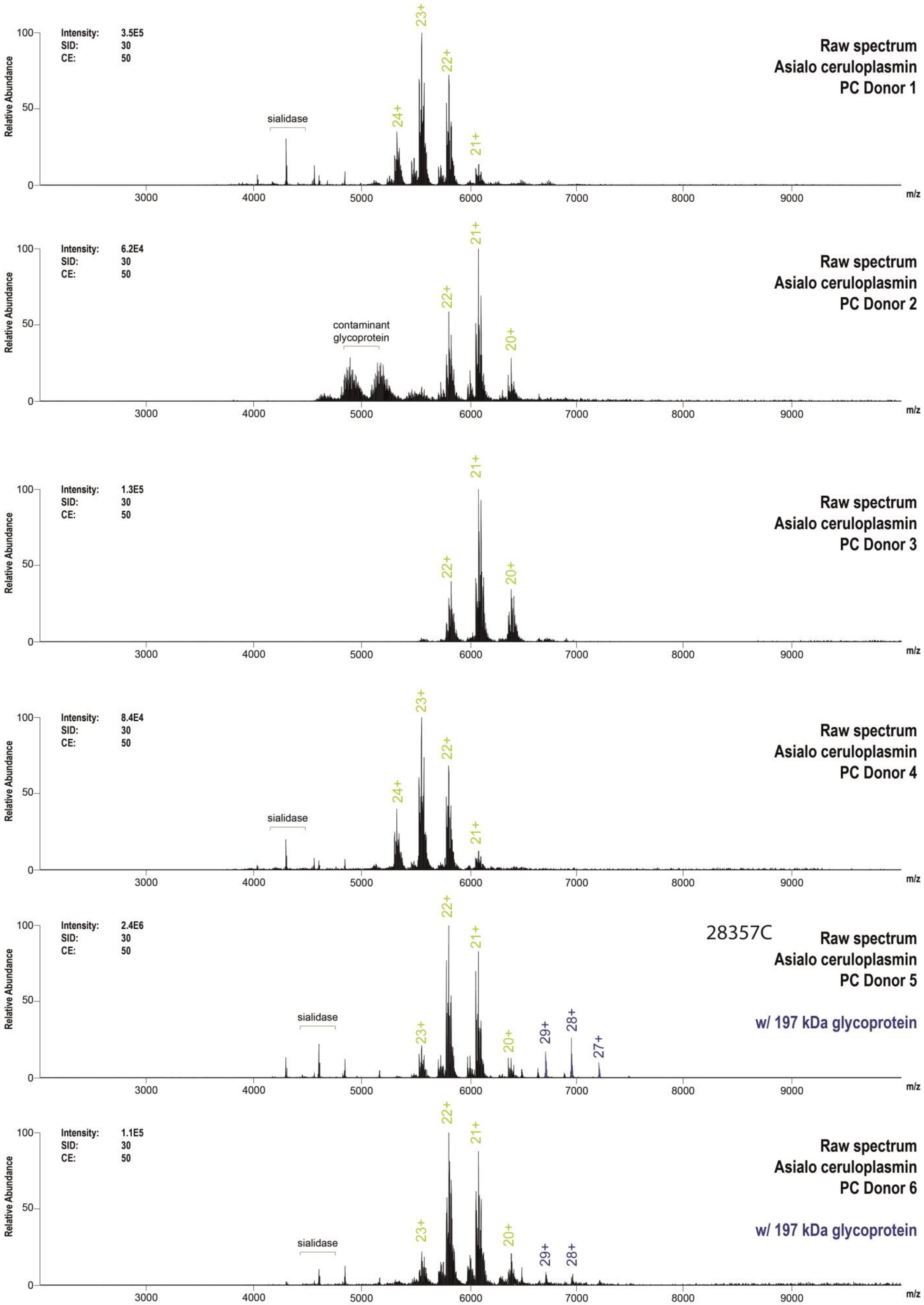

Intensity: 3.5E5
SID: 30
CE: 50
Raw spectrum
Asialo ceruloplasmin
PC Donor 1
sialidase
24+
23+
22+
21+
Relative Abundance
m/z
Intensity: 6.2E4
SID: 30
CE: 50
Raw spectrum
Asialo ceruloplasmin
PC Donor 2
contaminant
glycoprotein
22+
21+
20+
Intensity: 1.3E5
SID: 30
CE: 50
Raw spectrum
Asialo ceruloplasmin
PC Donor 3
22+
21+
20+
Intensity: 8.4E4
SID: 30
CE: 50
Raw spectrum
Asialo ceruloplasmin
PC Donor 4
sialidase
24+
23+
22+
21+
Intensity: 2.4E6
SID: 30
CE: 50
28357C
Raw spectrum
Asialo ceruloplasmin
PC Donor 5
w/ 197 kDa glycoprotein
sialidase
23+
22+
21+
20+
29+
28+
27+
Intensity: 1.1E5
SID: 30
CE: 50
Raw spectrum
Asialo ceruloplasmin
PC Donor 6
w/ 197 kDa glycoprotein
sialidase
23+
22+
21+
20+
29+
28+

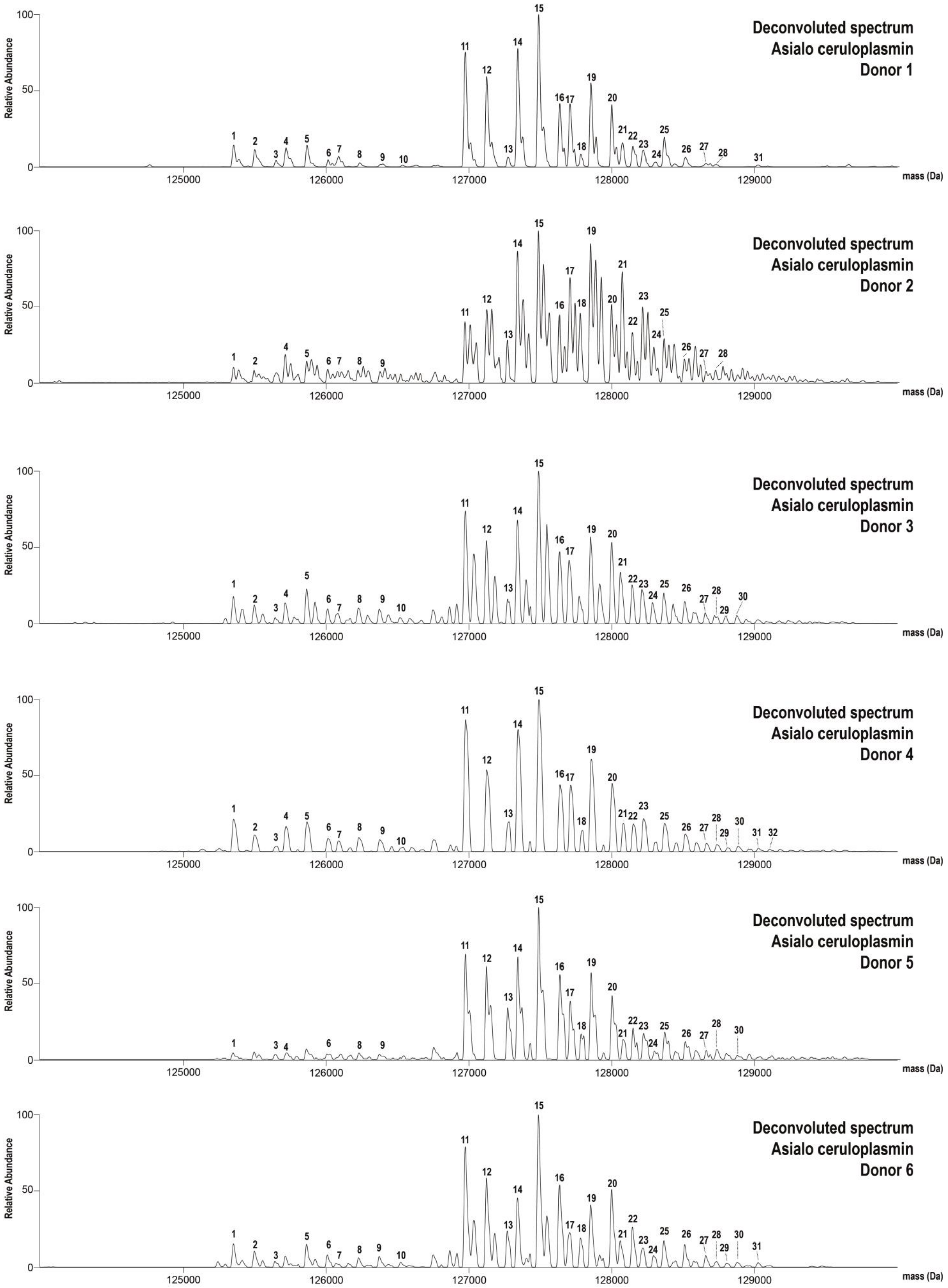
Deconvoluted spectrum
Asialo ceruloplasmin
Donor 1
Deconvoluted spectrum
Asialo ceruloplasmin
Donor 2
Deconvoluted spectrum
Asialo ceruloplasmin
Donor 3
Deconvoluted spectrum
Asialo ceruloplasmin
Donor 4
Deconvoluted spectrum
Asialo ceruloplasmin
Donor 5
Deconvoluted spectrum
Asialo ceruloplasmin
Donor 6
Relative Abundance
0
50
100
125000
126000
127000
128000
129000
mass (Da)

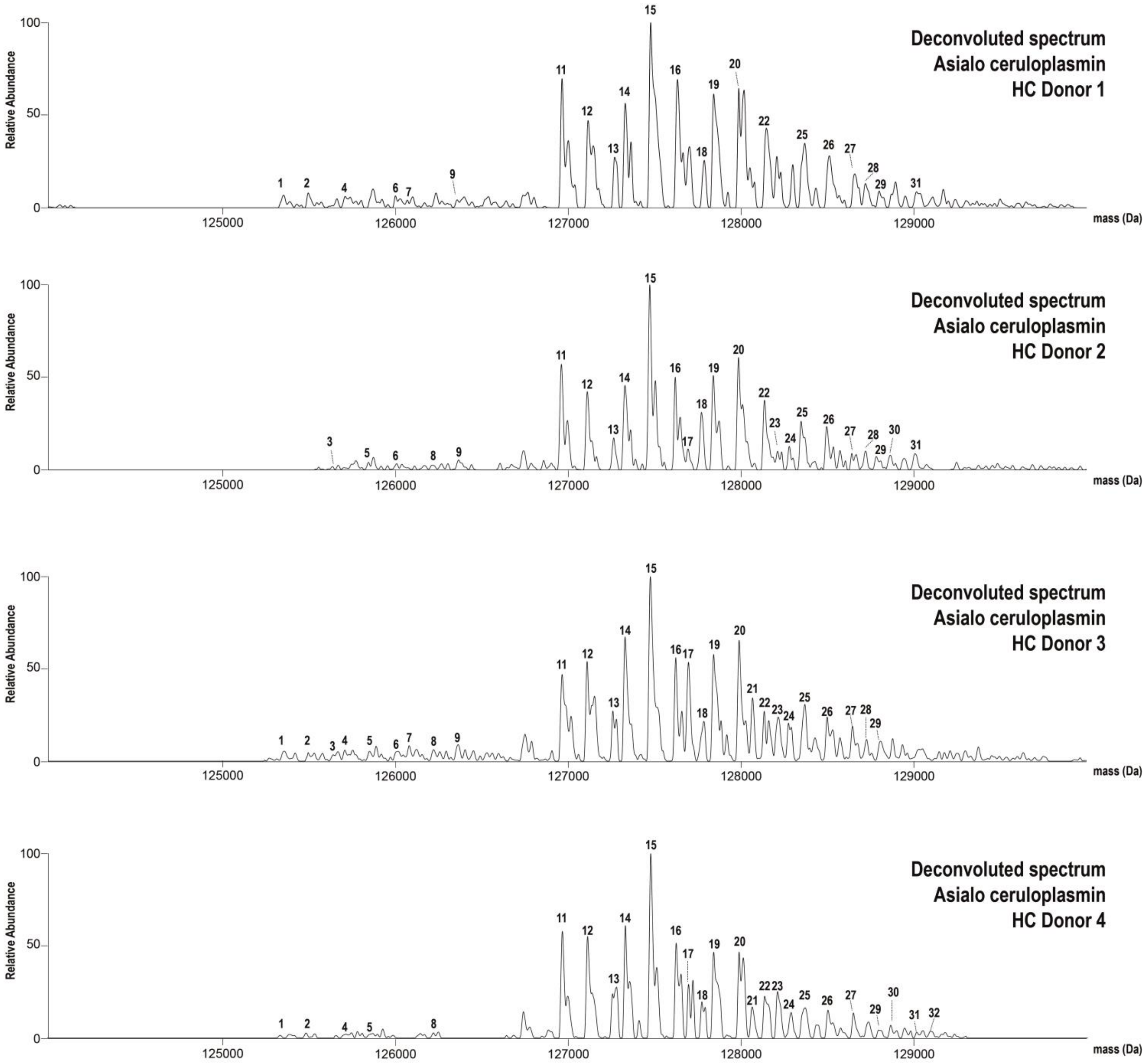
Deconvoluted spectrum
Asialo ceruloplasmin
HC Donor 1
Relative Abundance
100
50
0
125000
126000
127000
128000
129000
mass (Da)
1 2 4 6 7 9 11 12 13 14 15 16 18 19 20 22 25 26 27 28 29 31
Deconvoluted spectrum
Asialo ceruloplasmin
HC Donor 2
Relative Abundance
100
50
0
125000
126000
127000
128000
129000
mass (Da)
3 5 6 8 9 11 12 13 14 15 16 17 18 19 20 22 23 24 25 26 27 28 29 30 31
Deconvoluted spectrum
Asialo ceruloplasmin
HC Donor 3
Relative Abundance
100
50
0
125000
126000
127000
128000
129000
mass (Da)
1 2 3 4 5 6 7 8 9 11 12 13 14 15 16 17 18 19 20 21 22 23 24 25 26 27 28 29
Deconvoluted spectrum
Asialo ceruloplasmin
HC Donor 4
Relative Abundance
100
50
0
125000
126000
127000
128000
129000
mass (Da)
1 2 4 5 8 11 12 13 14 15 16 17 18 19 20 21 22 23 24 25 26 27 29 30 31 32

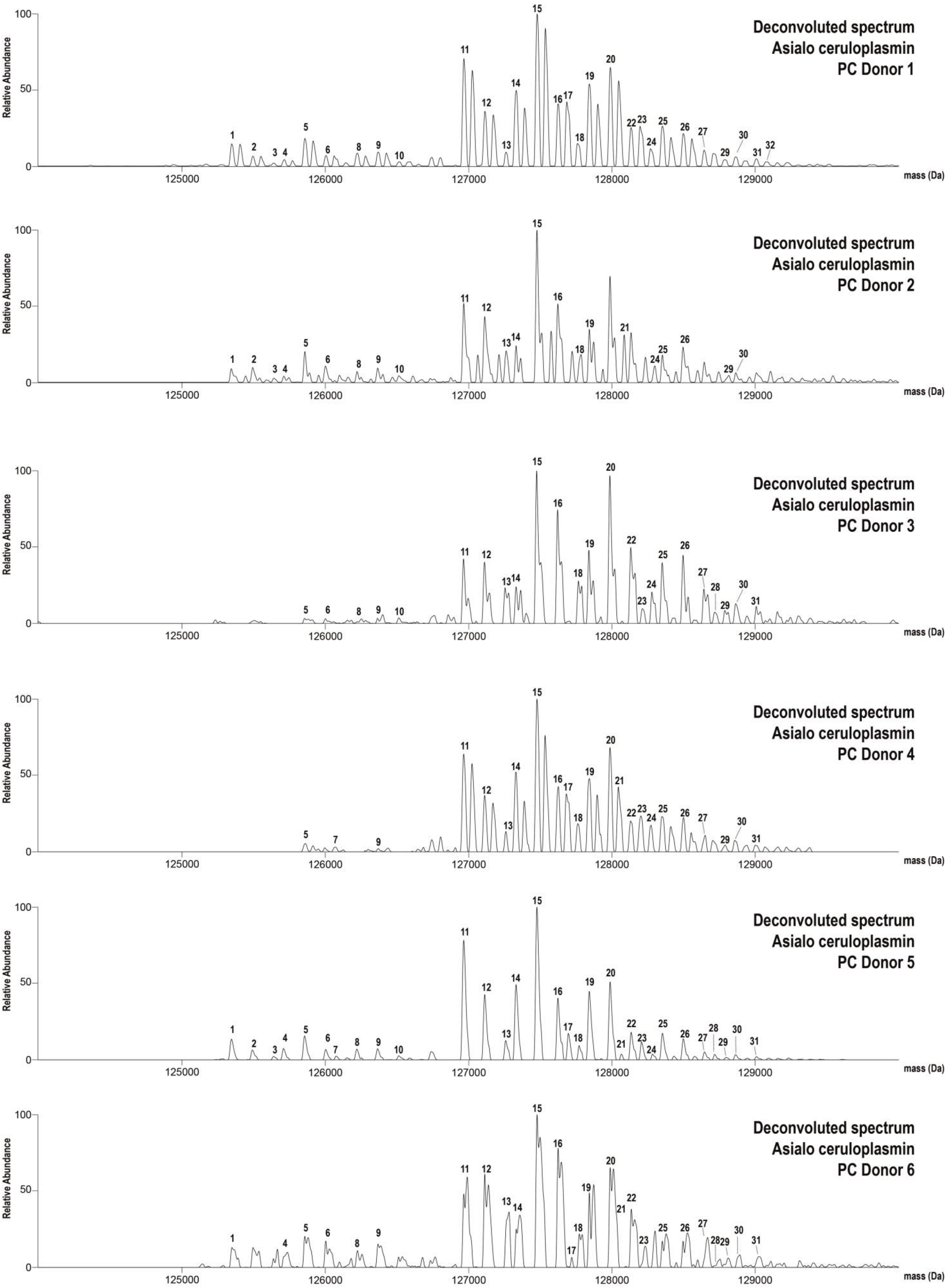
Deconvoluted spectrum
Asialo ceruloplasmin
PC Donor 1
Deconvoluted spectrum
Asialo ceruloplasmin
PC Donor 2
Deconvoluted spectrum
Asialo ceruloplasmin
PC Donor 3
Deconvoluted spectrum
Asialo ceruloplasmin
PC Donor 4
Deconvoluted spectrum
Asialo ceruloplasmin
PC Donor 5
Deconvoluted spectrum
Asialo ceruloplasmin
PC Donor 6
Relative Abundance
mass (Da)
0
50
100
125000
126000
127000
128000
129000
