# Supplementary material for: Charting the Proteoform Landscape of Serum Proteins in Individual Donors by High-Resolution Native Mass Spectrometry
Source: Anal Chem. 2022 Sep 8;94(37):12732–41. doi: 10.1021/acs.analchem.2c02215 (PMC9494300; doi:10.1021/acs.analchem.2c02215)

Supporting information for:

Charting the proteoform landscape of the serum proteins by high-resolution native mass spectrometry

Dario A. T. Cramer[1,2], Vojtech Franc[1,2], Tomislav Caval[1,2,3], Albert J. R. Heck[1,2 *].

[1]Biomolecular Mass Spectrometry and Proteomics, Bijvoet Center for Biomolecular Research and Utrecht Institute for Pharmaceutical Science, University of Utrecht, Padualaan 8, Utrecht, 3584 CH, The Netherlands

[2]Netherlands Proteomics Centre, University of Utrecht, Padualaan 8, Utrecht, 3584 CH, The Netherlands

[3]current address, Stanford

Corresponding Author

[*]e-mail: a.j.r.heck@uu.nl, tel: +31 - 302536797

This file contains:

Supplement S2

Native MS spectra, raw and deconvoluted, of all proteins observed in the study

1, 2: ***P1, Hemopexin***

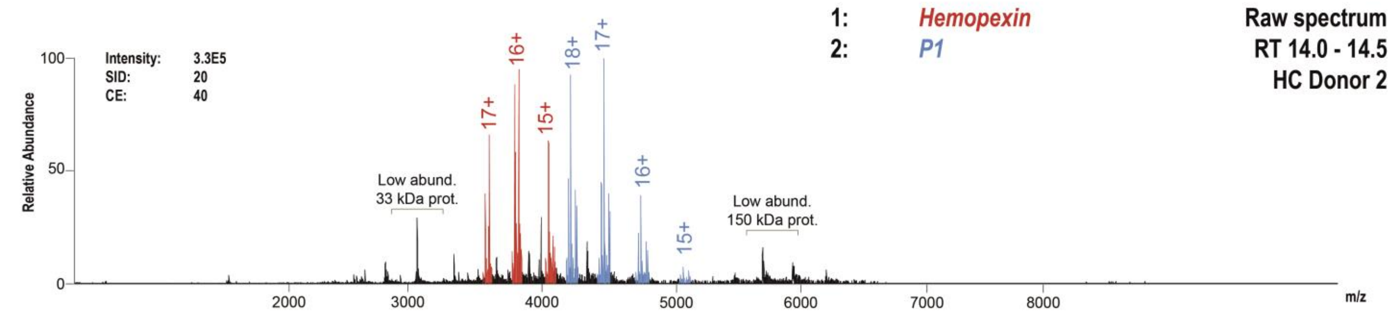

1: ***Hemopexin***

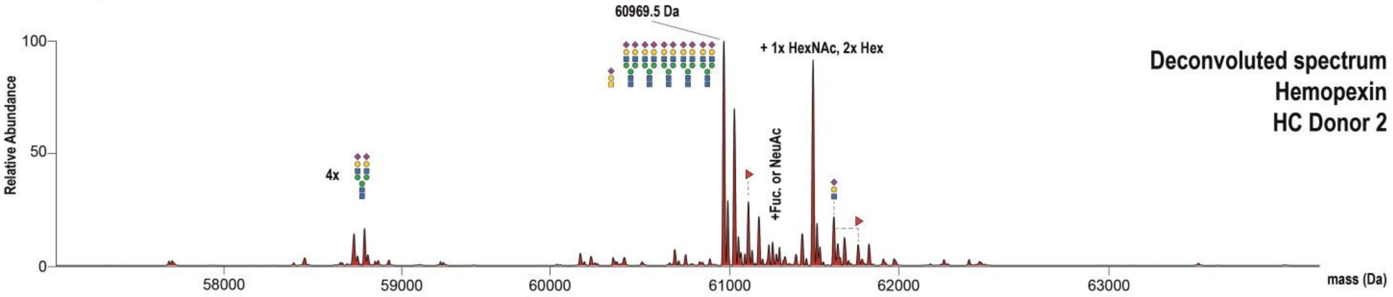

2: ***P1***

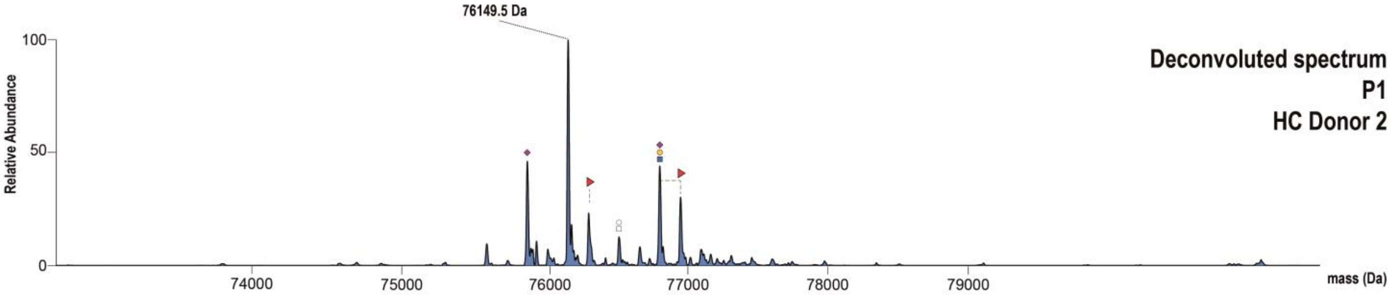

3: ***Plasminogen***

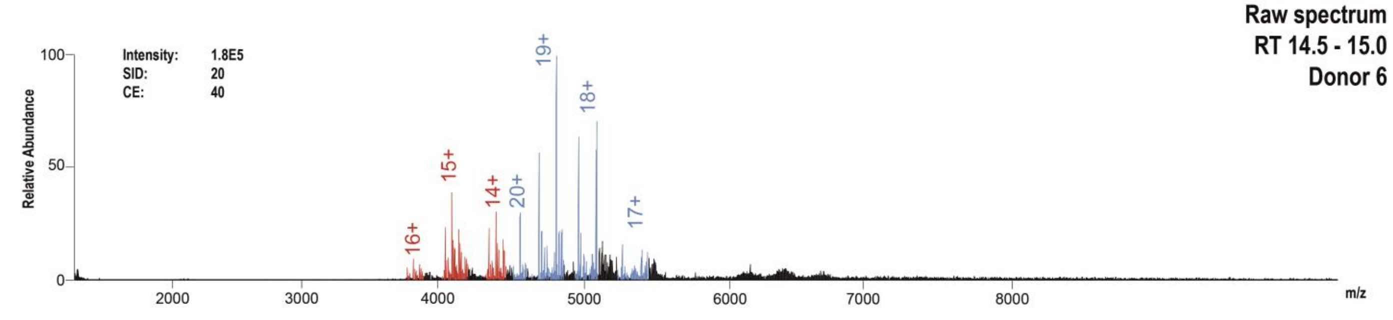

3: ***Plasminogen***

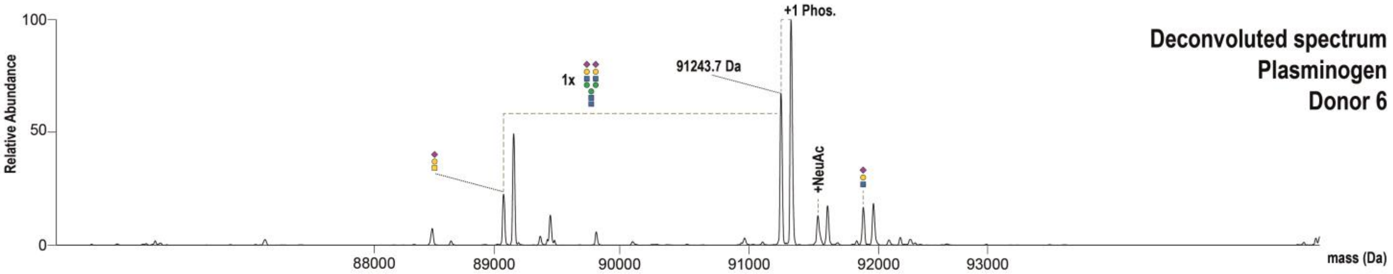

4: ***Alpha-1-antitrypsin***

5: ***Albumin***

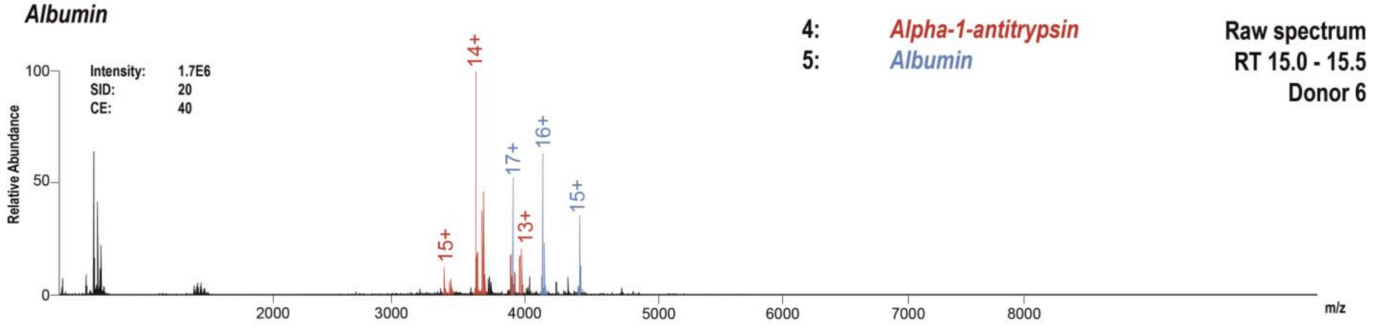

4: ***Alpha-1-antitrypsin (m1v)***

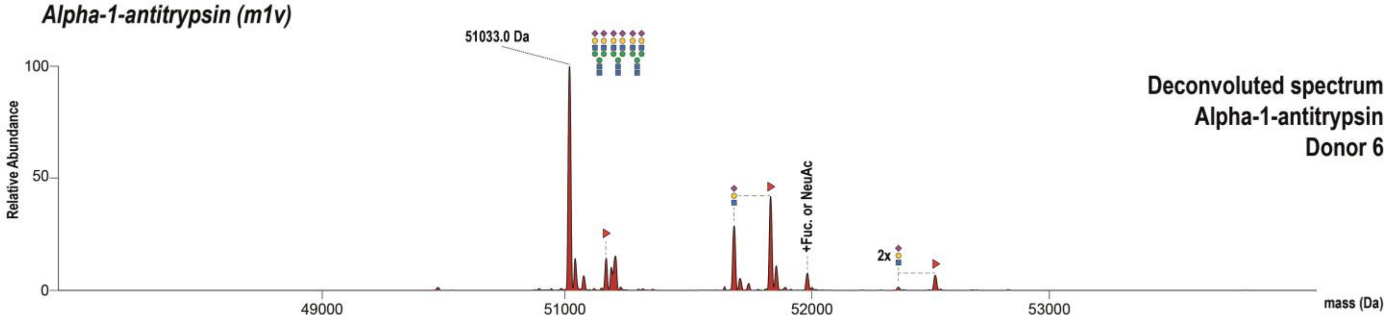

5: ***Albumin***

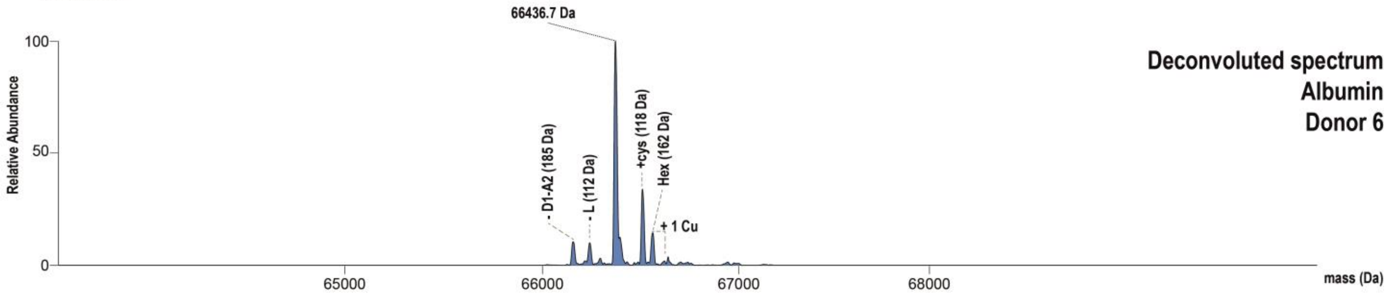

6: *P2*

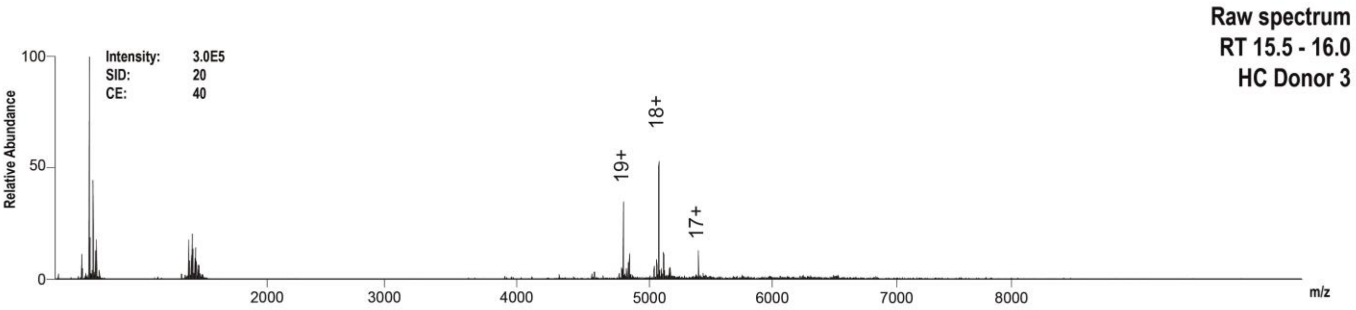

6: *P2*

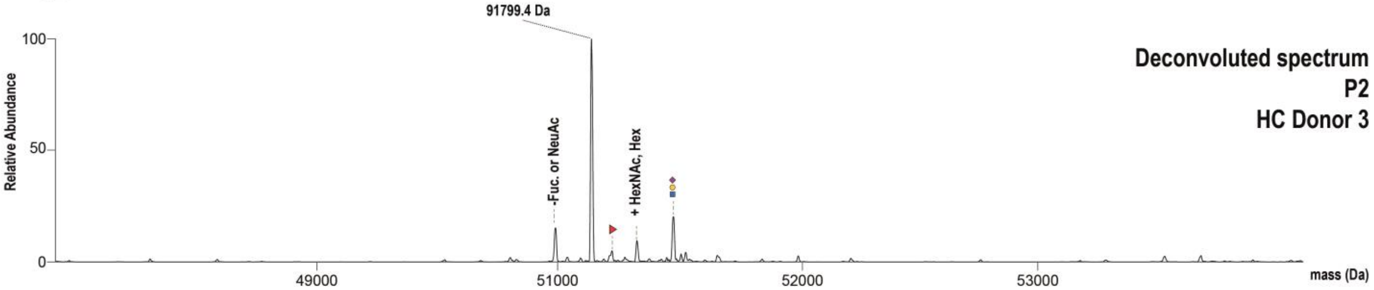

7: *P3*

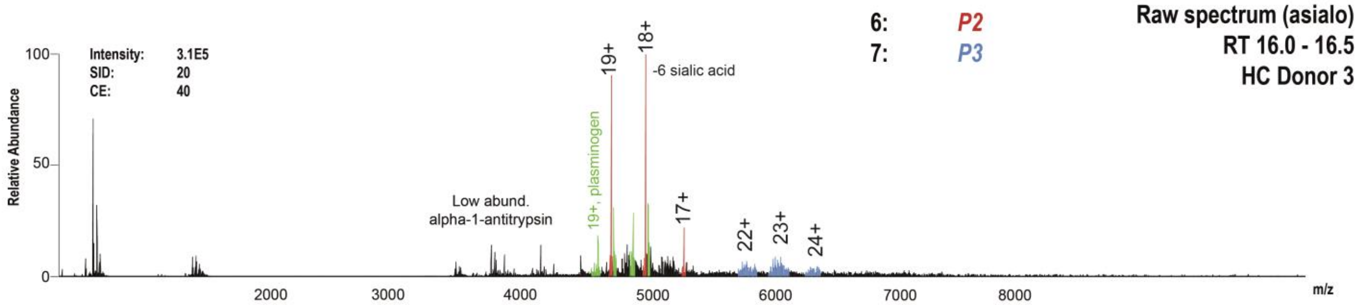

7: *P3*

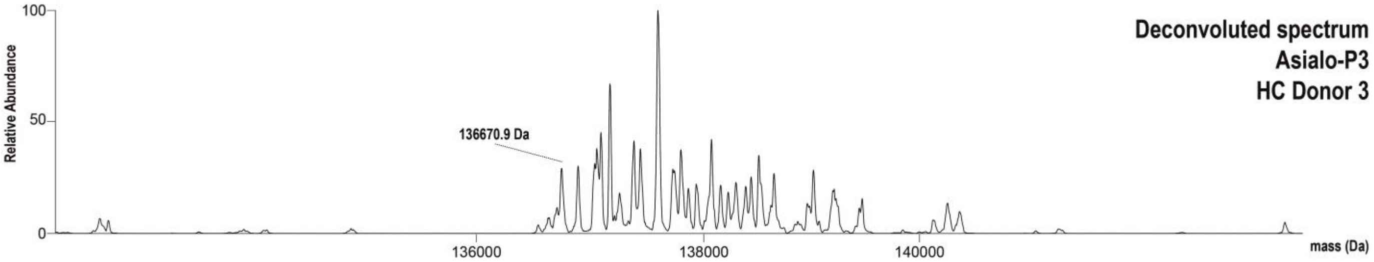

8: ***P4***
9: ***P5***
10: ***P6***
11: ***P7***

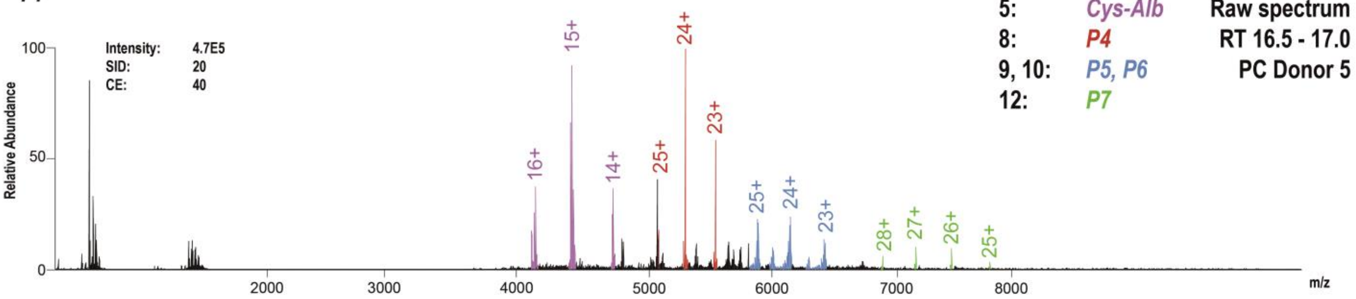

9: ***P4***

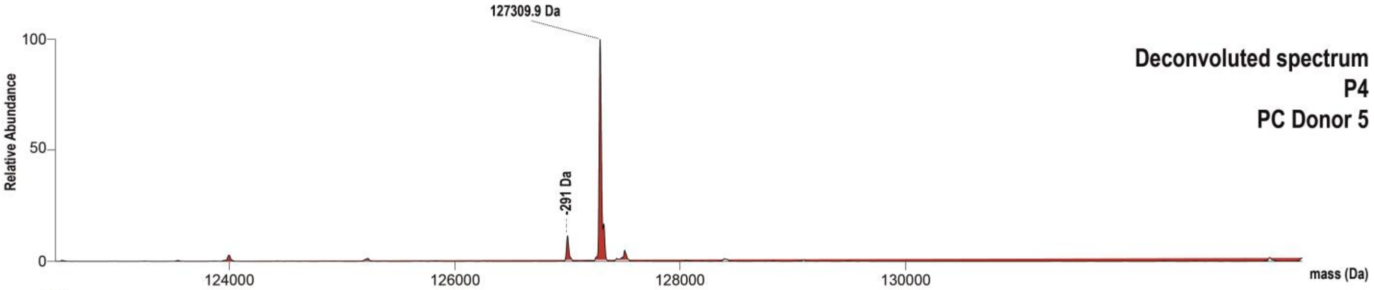

10: ***P5***

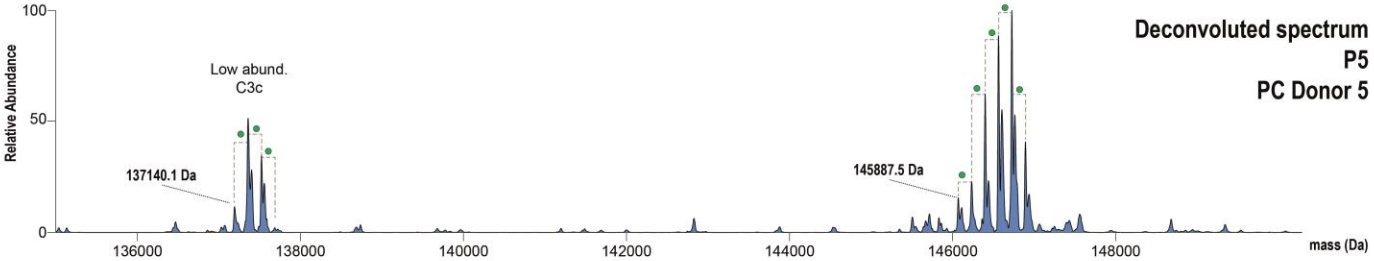

11: ***P6***

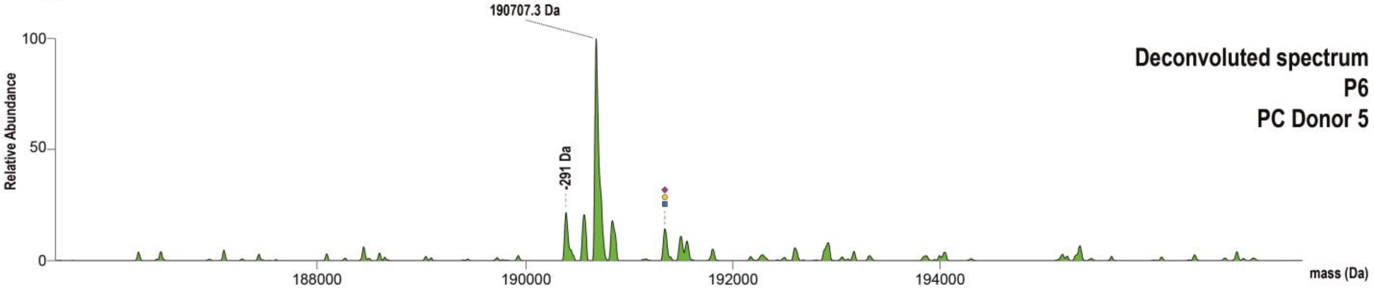

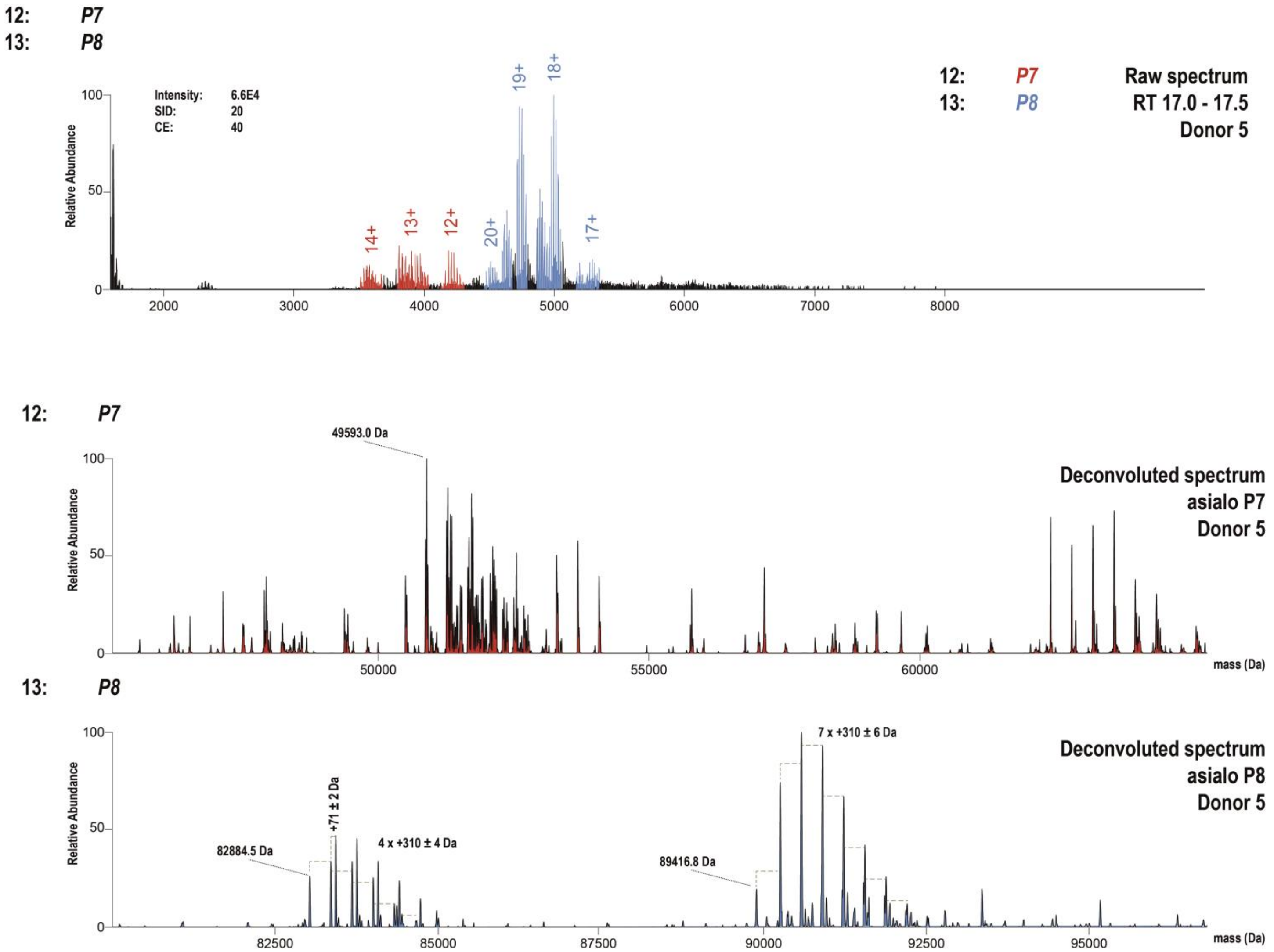
12: P7
13: P8
Intensity: 6.6E4
SID: 20
CE: 40
12: P7
13: P8
Raw spectrum
RT 17.0 - 17.5
Donor 5
14+
13+
12+
20+
19+
18+
17+
Relative Abundance
100
50
0
2000
3000
4000
5000
6000
7000
8000
12: P7
49593.0 Da
Deconvoluted spectrum
asialo P7
Donor 5
50000
55000
60000
mass (Da)
13: P8
82884.5 Da
+71 ± 2 Da
4 x +310 ± 4 Da
89416.8 Da
7 x +310 ± 6 Da
Deconvoluted spectrum
asialo P8
Donor 5
82500
85000
87500
90000
92500
95000
mass (Da)

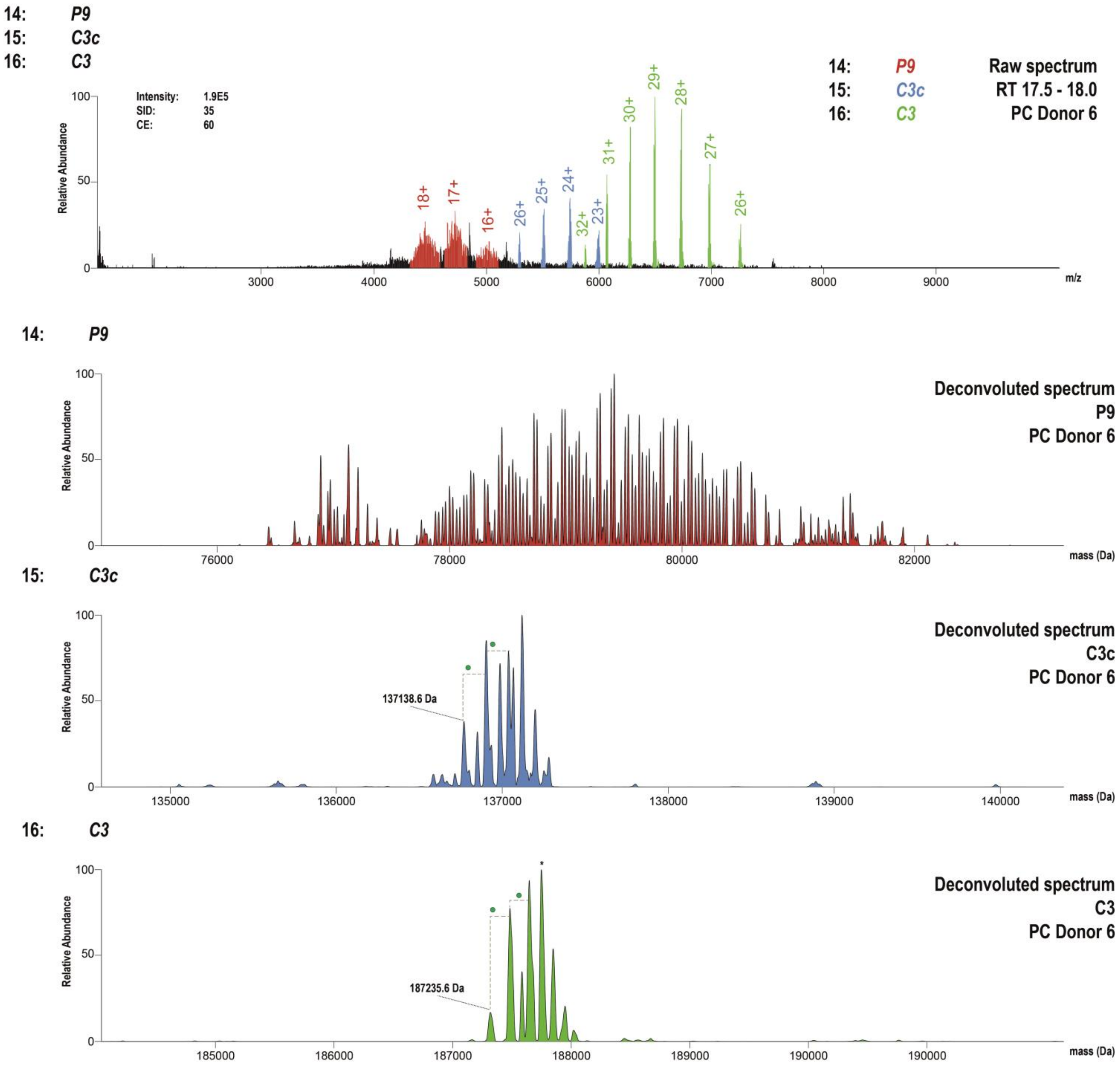

14: P9
15: C3c
16: C3
Intensity: 1.9E5
SID: 35
CE: 60
14: P9 Raw spectrum
15: C3c RT 17.5 - 18.0
16: C3 PC Donor 6
Relative Abundance
m/z
18+
17+
16+
26+
25+
24+
32+
23+
31+
30+
29+
28+
27+
26+
14: P9
Deconvoluted spectrum
P9
PC Donor 6
mass (Da)
15: C3c
Deconvoluted spectrum
C3c
PC Donor 6
137138.6 Da
16: C3
Deconvoluted spectrum
C3
PC Donor 6
187235.6 Da

17: ***Alpha-1-acid glycoprotein***

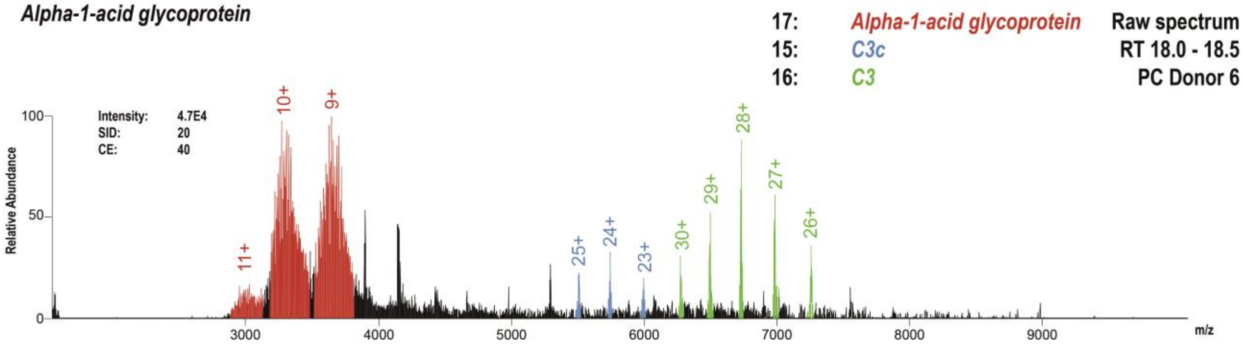

17: ***Alpha-1-acid glycoprotein***

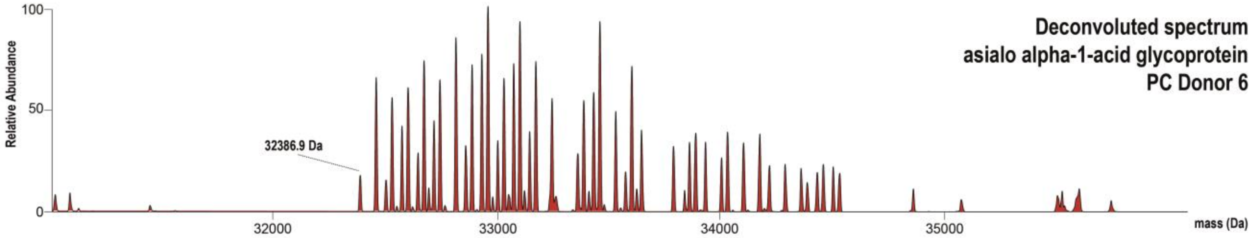

18: ***P10***

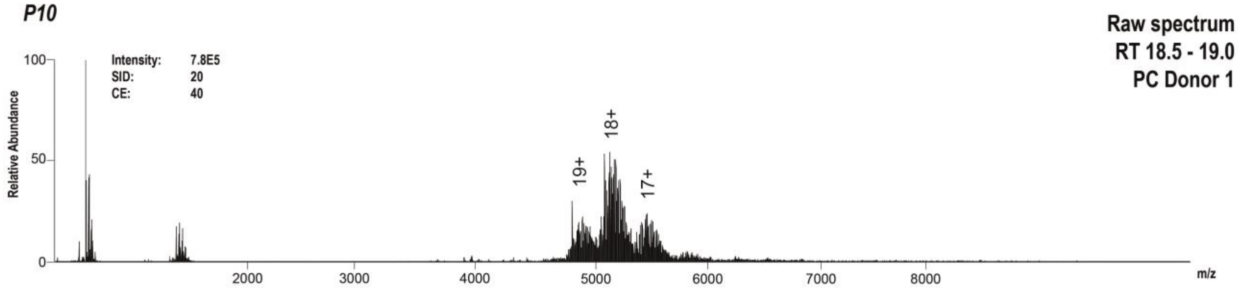

18: ***P10***

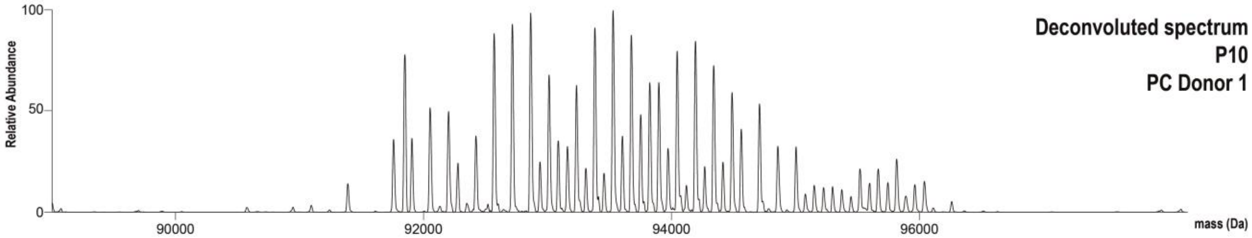

19: ***Fetuin***

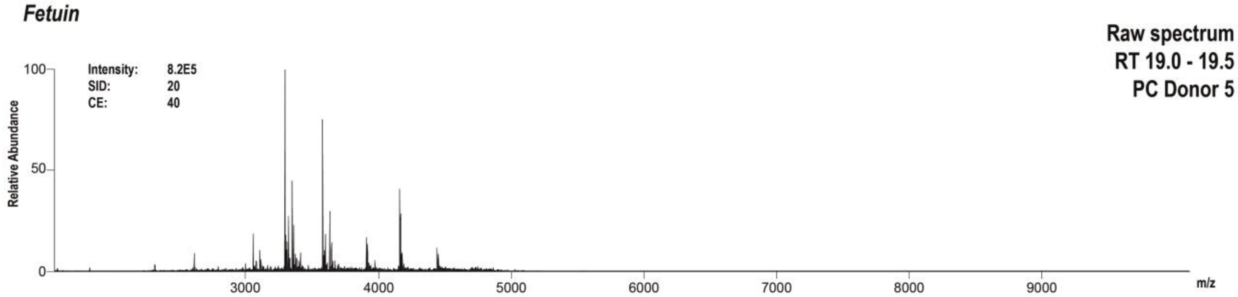

19: ***Fetuin***

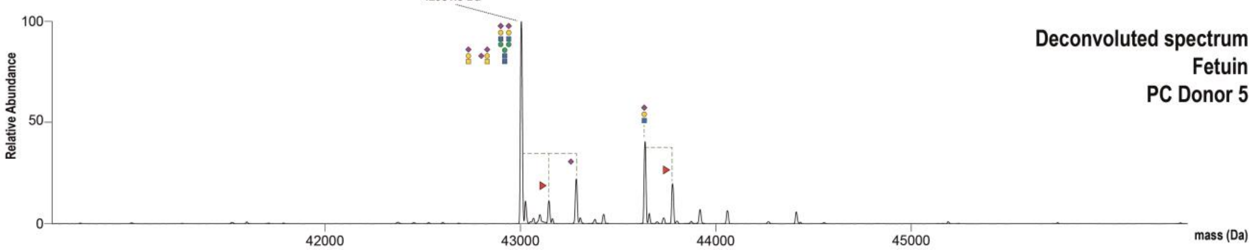

19: ***Ceruloplasmin***

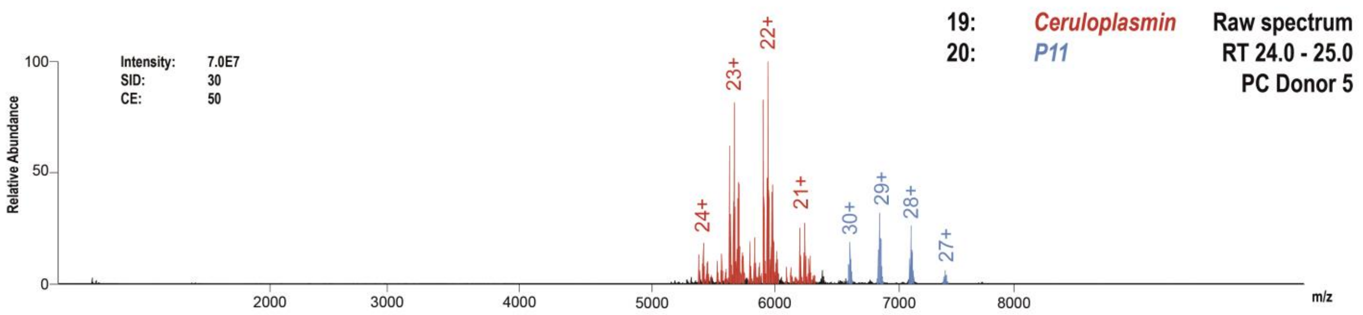

19: ***Ceruloplasmin***

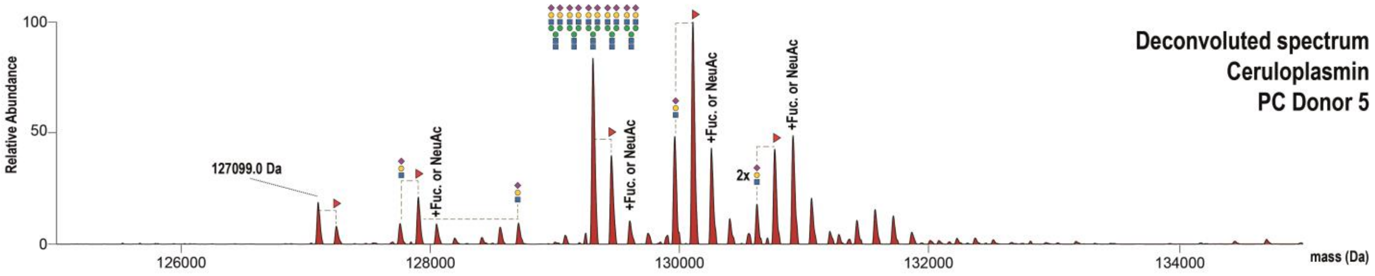

20: ***P11***

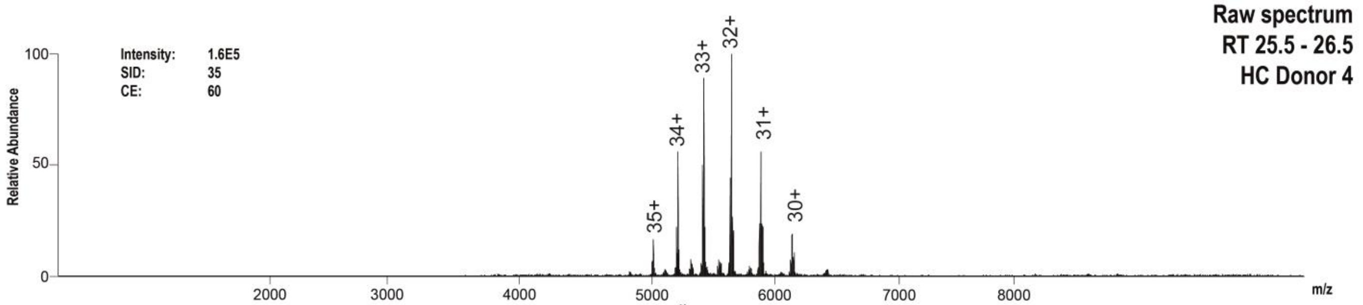

20: ***P11***

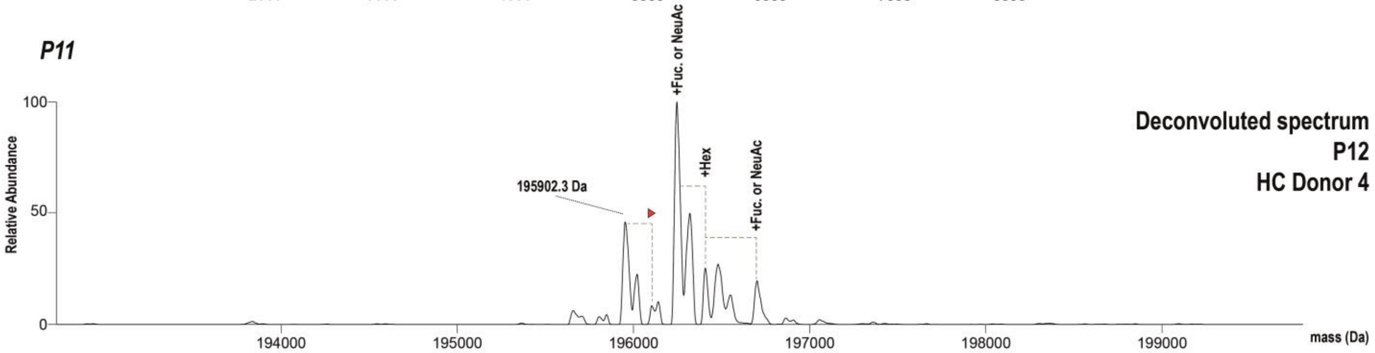

Supplement: Supplementary file 4 — ac2c02215_si_004.pdf [file ac2c02215_si_004.pdf]
